# Supplementary material for: A mathematical, classical stratification modeling approach to disentangling the impact of weather on infectious diseases: A case study using spatio-temporally disaggregated Campylobacter surveillance data for England and Wales
Source: PLoS Comput Biol. 2024 Jan 18;20(1):e1011714. doi: 10.1371/journal.pcbi.1011714 (PMC10796013; doi:10.1371/journal.pcbi.1011714)
Supplement: S1 Text — Removing Reporting Delays and the effect of Incubation Period. Correlations among the weather variables and their distributions. Validation with Agent Based Models. Patterns in conditional incidence according to different weather variables (two weather factors simultaneously). Different Ways to Visualize conditional incidence (three weather factors simultaneously). Patterns in conditional incidence according to different weather variables (four weather factors simultaneously). Patterns in conditional incidence according to maximum air temperature and relative humidity for different periods of the year. Incidence of campylobacteriosis cases when the weather variables are averaged over different time-lags (three weather factors simultaneously). Seasonal patterns for daily Campylobacter cases using only one predictor. Seasonal patterns for daily Campylobacter cases using only two predictors. Predictions using rainfall, instead of relative humidity, as predictor. (PDF) [file pcbi.1011714.s001.pdf]

A mathematical, classical stratification modeling approach to disentangling the impact of weather on infectious diseases: a case study using spatio-temporally disaggregated *Campylobacter* surveillance data for England and Wales

S1 Text

A Regional structure of UK Health Security Agency, diagnostic laboratories and their catchment areas.

As of 2020, UKHSA is structured into a national centre, 4 regions and 8 centres plus London, (the latter is an integrated region-centre) [1], with 416 diagnostic laboratories in England and Wales serving patients residing within a local catchment area (see Fig. 3 in [2] and Fig. A).

There are no official records, however, of the boundary of the catchment areas, which can also change in time. The algorithm to estimate the population living within each catchment area was thus based on the census data from 1990 to 2015 and 472, 218 records of campylobacteriosis (corresponding to the only cases for which the domestic address of the patient were known) to approximate the geographical boundaries of the catchment areas.

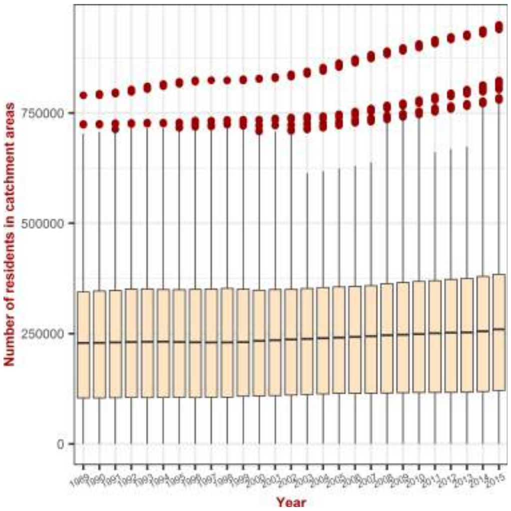

Fig A. Boxplot showing the number of residents in all catchment areas from 1989 to 2015. The outliers correspond to the following postal codes: 'LE1 5WW' (served by Leicester Royal Infirmary - NHS, Leicester, Leicestershire); 'CV1 4FH' (Coventry NHS Walk-In Centre, Coventry, West Midlands); 'CV34 5BW' (Warwick Hospital - NHS, Warwick, Warwickshire); 'GU16 7UJ' (Frimley Park Hospital - NHS, Camberley, Surrey); 'NG7 2UH' (Nottingham University Hospitals NHS Trust, Nottingham, Nottinghamshire); 'AL7 4HQ' (Queen Elizabeth I I Hospital - NHS, Welwyn Garden City, Hertfordshire); 'OL1 2JH' (Royal Oldham Hospital - NHS, Oldham, Lancashire).

B Removing Reporting Delays and the effect of Incubation Period.

Regardless of the causal pathway of infection, the impact of the weather factors on the occurrence of campylobacteriosis is not instantaneous. This introduces a time-lag between the time when these weather factors start to have an effect on the risk of infection and when the infection occurs. Following the general arguments discussed in Lo Iacono *et al.* [3], it is helpful to separate the main sources of time-lags: i) the aetiological time-lag (driven by a variety of environmental, ecological, epidemiological and socio-economic factors); ii) incubation period (patient's physiology, source of infection); and iii) total reporting delay (patient behaviour and other factors such as easy of access to diagnostic facilities, efficiency of the reporting system etc.). Here we

adjusted the data to remove the incubation period and the reporting delay. The distributions of the incubation period (figure B) and reporting delay were assumed to be log-normal [4, 5] with location parameter, μ, and the scale parameter, σ inferred by the mean, m, and standard deviation, s, of the observed data of Horn and Lake [6] for the incubation period. Delay in reporting was assumed to follow a uniform distribution between $1-4$ days, based on informed opinion of one of the authors (GN).

$$\mu = \ln\left[\frac{m}{\sqrt{1+\frac{s^2}{m^2}}}\right]; \quad \sigma = \sqrt{\ln\left[1+\frac{s^2}{m^2}\right]}.$$

Thus for each *Campylobacter* case, we numerically generated a random value of the incubation period, t_{Incub}, and of the reporting delay, t_{Det}, drawn from the corresponding distributions. The sum of these two random numbers corresponded to the overall delay since infection. Each *Campylobacter* case was then assumed to occur at date $t_{Occur} = t_{Reported} - t_{Incub} - t_{Det}$, where $t_{Reported}$ is the date when the specimen was received by the diagnostic laboratories.

Reported and adjusted *Campylobacter* cases in England and Wales

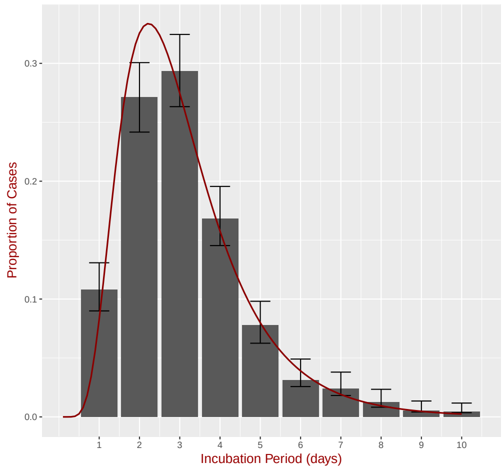

Fig B. Distribution of the incubation period based on the data of [6]. The bars represent the proportion of campylobacteriosis cases from 832 cases associated with outbreaks with known exposure events, the black lines represent the 2.5^{th} and 97.5^{th} percentile bootstrap intervals of the proportion; the solid red line represents the fitted log-normal distribution with the same mean (3.14 days) and standard deviation (1.59 days) of the observed cases.

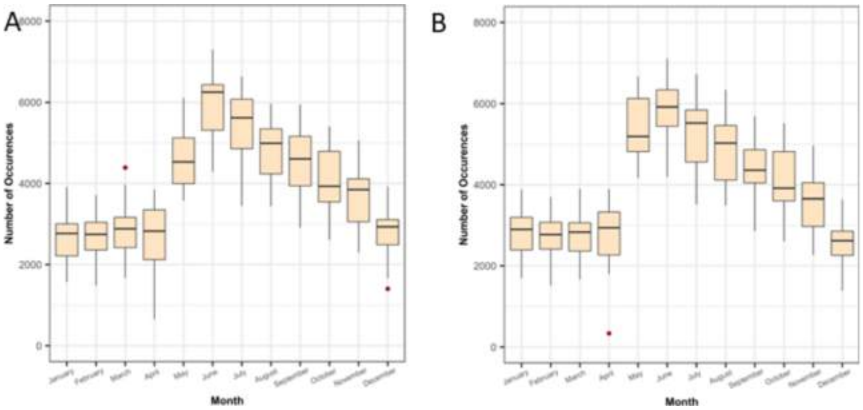

Fig C. Boxplot showing campylobacteriosis in England and Wales from 1990 to 2009. A) reported cases and B) corrected cases by removing incubation period and reporting delay.

C Correlations among the weather variables and their distributions.

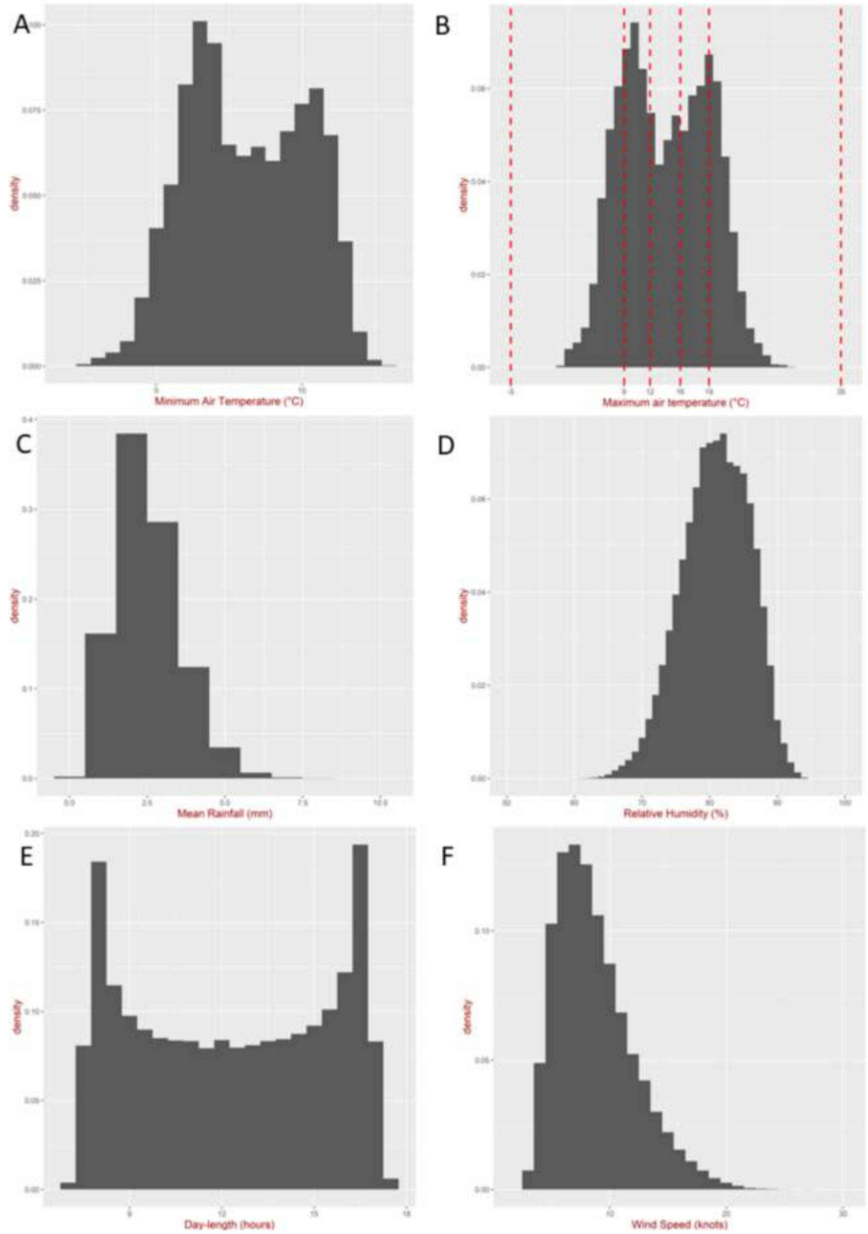

Fig D. Histograms showing the distribution of the weather variables estimated at all catchment areas from 1990 to 2009. The y-axis represents the density of points in bin (number of points divided the size of the bin), scaled to integrate to 1. Weather variables are averaged over the past 14 day. As an example, the vertical, red lines in panel B show the location of the quantiles values (rounded to the nearest integer for visual purposes), *i.e.* the cut points dividing the range of maximum air temperature in intervals of variable size so that the number of observations in each bin are equal (here 20% of total observations in each bin).

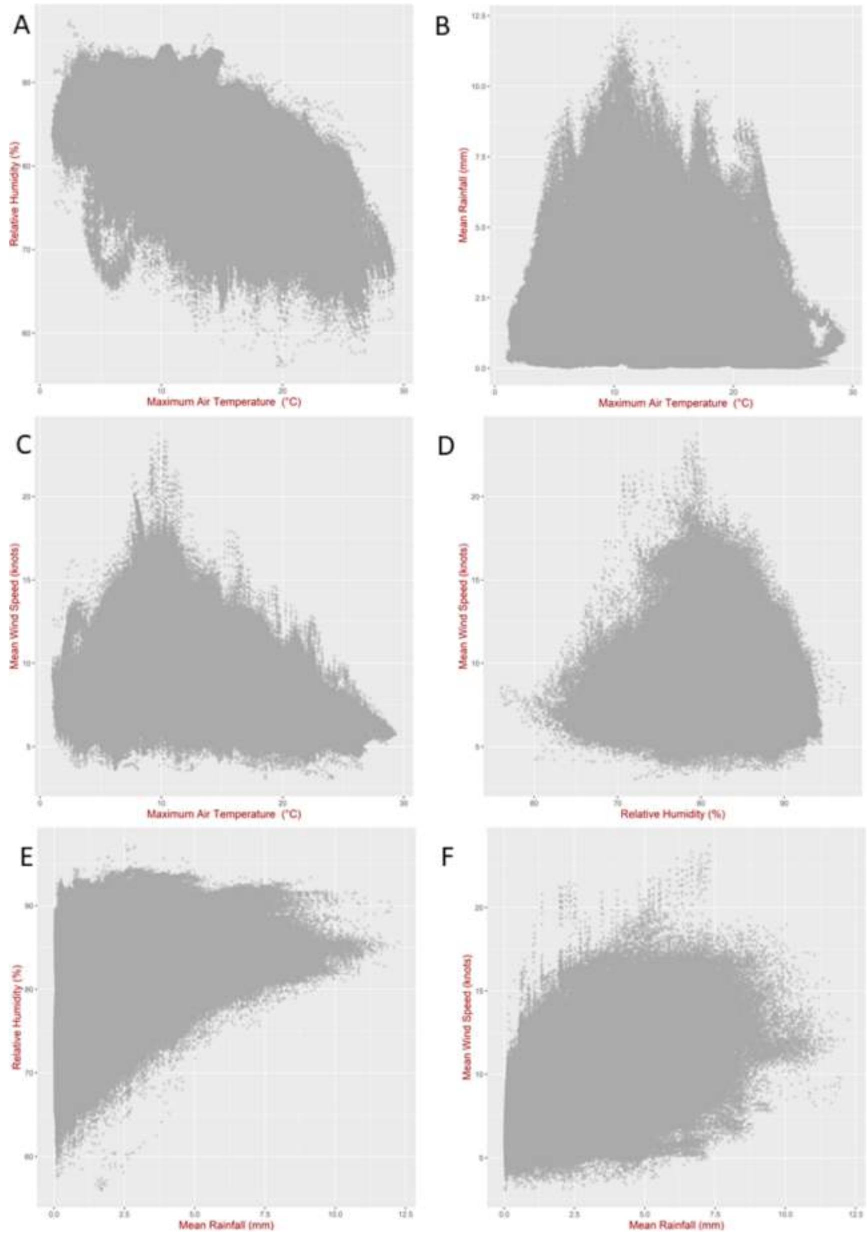

Fig E. Continued on next page

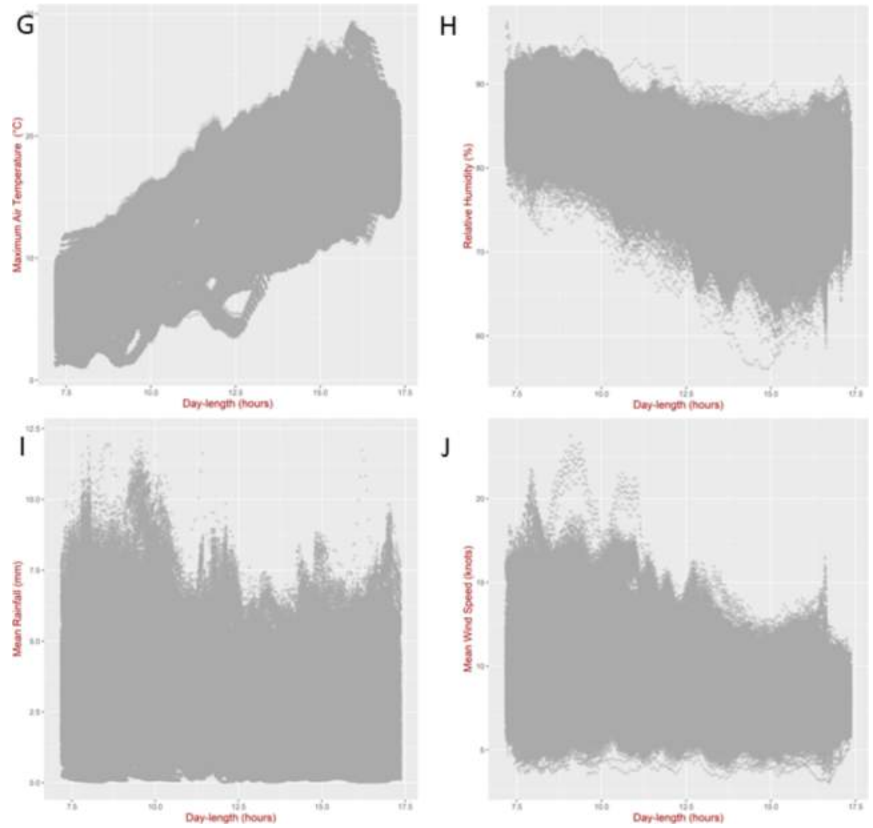

Fig E. Scatter plots showing correlation between weather variables. Each dot represent the values of two weather variables, averaged over the past 14 days, for all catchment areas from 1990 to 2009.

D Validation with Agent Based Models.

The approach has been validated by comparing it with synthetic disease data generated by an Agent Based Model (ABM). Namely, disease data are generated randomly *via* a Poisson process with two different rates λ and time-lag one day. In the first case the rate depends only on maximum air temperature T in a triangular shape according to the function:

$$\lambda = \begin{cases} 10^{-5}(15 + \overline{T^{max}_{t,\mathbf{x}}}^{p}) & \text{if } \overline{T^{max}_{t,\mathbf{x}}}^{p} \leq 13°C \\ 10^{-5}(41 - \overline{T^{max}_{t,\mathbf{x}}}^{p}) & \text{if } \overline{T^{max}_{t,\mathbf{x}}}^{p} > 13°C \end{cases} \tag{A}$$

in the second case the rate depend on maximum air temperature T and relative humidity RH as:

$$\lambda = \begin{cases} 10^{-5}\left[\overline{RH_{t,\mathbf{x}}}^{p}\right]^2 (15 + \overline{T^{max}_{t,\mathbf{x}}}^{p}) & \text{if } \overline{T^{max}_{t,\mathbf{x}}}^{p} \leq 13°C \\ 10^{-5}\left[\overline{RH_{t,\mathbf{x}}}^{p}\right]^2 (41 - \overline{T^{max}_{t,\mathbf{x}}}^{p}) & \text{if } \overline{T^{max}_{t,\mathbf{x}}}^{p} > 13°C \end{cases} \tag{B}$$

where for maximum air temperature and relative humidity we used data from 1990 to 2015 recorded in the catchment area associated with the reference laboratory in Welwyn Garden City in England, and averaged over the previous day (i.e. x corresponds to postcode $AL7$ $4HQ$ and $p = 1$). This choice, as well as the choice of the shape for the conditional incidence, the related parameters (*i.e.* the $3°C$ threshold) is deliberately arbitrary. The purpose of this exercise is to show that the method and its numerical implementation is able to correctly identify arbitrary shapes of the conditional incidence.

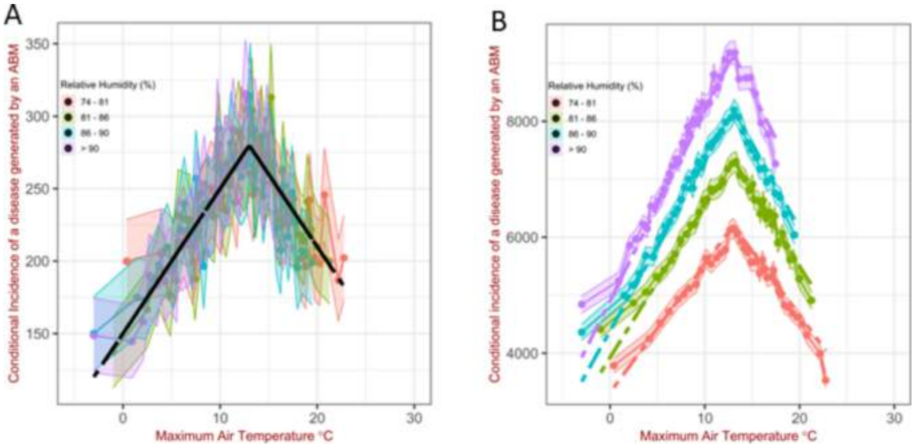

Fig F. Comparison of model outputs *vs* patterns of a synthetic disease data (not campylobacteriosis) generated by an ABM. A) Disease cases per $1,000,000$ per day conditioned to maximum air temperature and relative humidity recorded the catchment area associated with the reference laboratory in Welwyn Garden City, England. The synthetic disease is generated by a Poisson process with rate depending only on maximum air temperature T in a triangular fashion represented by the dashed black line and equation (A). Since relative humidity is not an explanatory variable all the profiles for the conditional incidence collapse to the same curve. B) As in A), but the synthetic disease is generated by a Poisson process with rate depending on both maximum air temperature T and relative humidity in a triangular fashion represented by the dashed lines and equation (B). Data were averaged over the previous day. The shaded area shows the 95% confidence intervals for the Poisson means using the normal approximation (*i.e.* average counts $\pm\, 1.96\sqrt{(\text{average counts/sample size})}$). Data divided by quantiles.

As shown in Fig. F the conditional incidence approach correctly estimated the conditional incidence of the disease generated by the ABM.

From the conditional incidence, we then reconstructed the time-series of synthetic disease cases as explained above. As the time-lag is, in general, not known *a priori*, we considered two situations: i) when the assumed time-lag was the same as in the ABM and ii) when it was different (30 days). As Fig. G shows, the reconstructed time-series closely mirrors the cases generated by the ABM when the chosen time-lag is the same as in the ABM. In contrast when there is a mismatch in the time-lags the reconstructed time-series shows a discrepancy with the cases from the ABM. This indicates that the actual time-lag in campylobacteriosis can be inferred by comparing the reconstructed time-series for different time-lags with real data and selecting the value that results in the best fit.

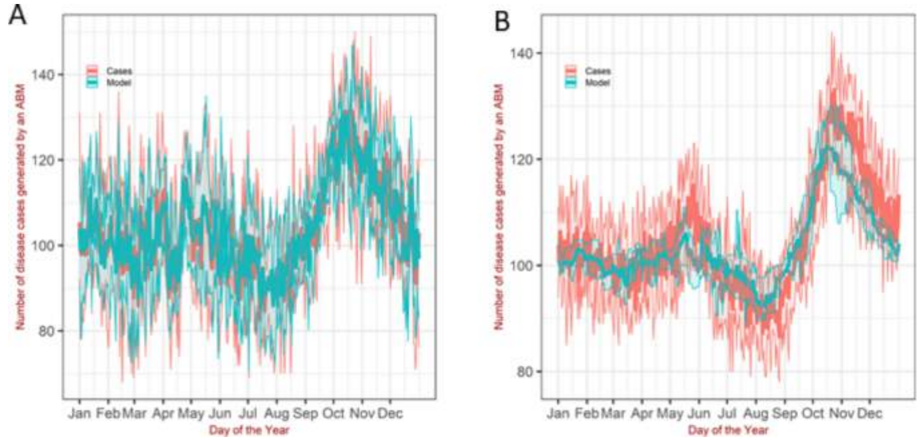

Fig G. Reconstruction of the time-series of synthetic disease cases (not campylobacteriosis) compared with the cases independently generated by the ABM according to equation (B). Cases aggregated by time of the year and averaged over 19 years. The shaded area represents the 25% and 75% quantiles. A) Assumed time-lag the same as used in the ABM. B) Assumed time-lag (30 days) different from the one used in the ABM (1 day).

E Patterns in conditional incidence according to different weather variables (two weather factors simultaneously).

Figure H shows relationships between the conditional incidence of campylobacteriosis cases *vs* maximum air temperature T conditional to i) relative humidity, ii) rainfall, iii) mean wind speed and iv) day-length. A similar analysis was done using minimum air temperature and the difference between maximum and minimum air temperature (Figs I, J). As the predictions did not qualitatively change, we continued using maximum air temperature for the rest of the analysis.

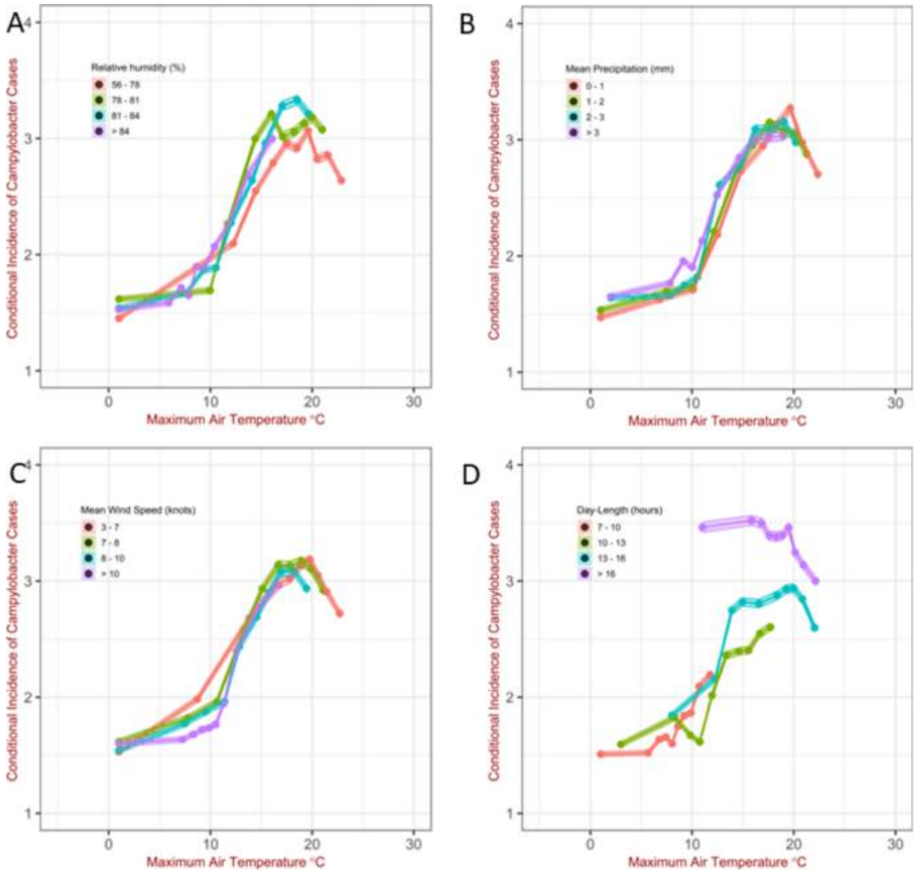

Fig H. Campylobacteriosis cases per $1,000,000$ per day conditioned to a particular value of maximum air temperature and relative humidity (A), maximum air temperature and rainfall (B), maximum air temperature and cumulative rainfall (C), maximum air temperature and mean wind speed (D). Data were averaged over the past 14 days. The shaded area shows the 95% confidence intervals for the Poisson means using the normal approximation (*i.e.* average counts $\pm\, 1.96\sqrt{(\text{average counts/sample size})}$). Data divided by quantiles to ensure the same number of observation in each bin.

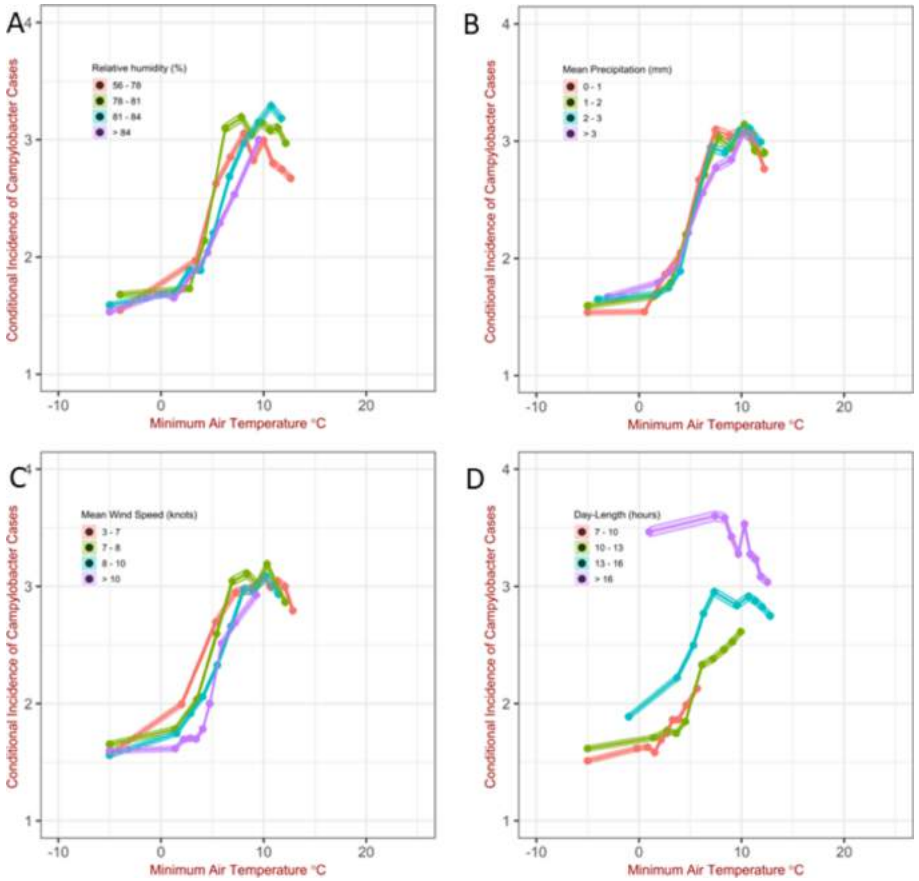

Fig I. Campylobacteriosis cases per 1,000,000 per day *vs* minimum air temperature rather than maximum air temperature (compare with figure H).

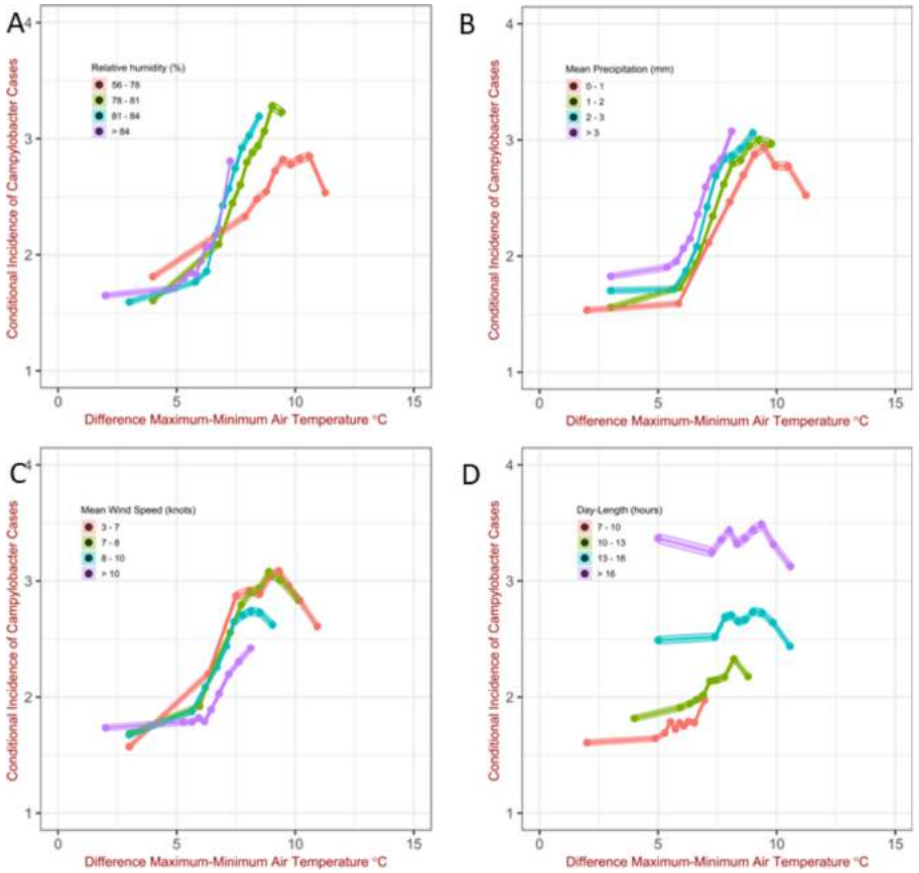

Fig J. Campylobacteriosis cases per 1, 000, 000 per day *vs* the difference between maximum and minimum air temperature rather than maximum air temperature (compare with figure H).

F Different Ways to Visualize conditional incidence (three weather factors simultaneously).

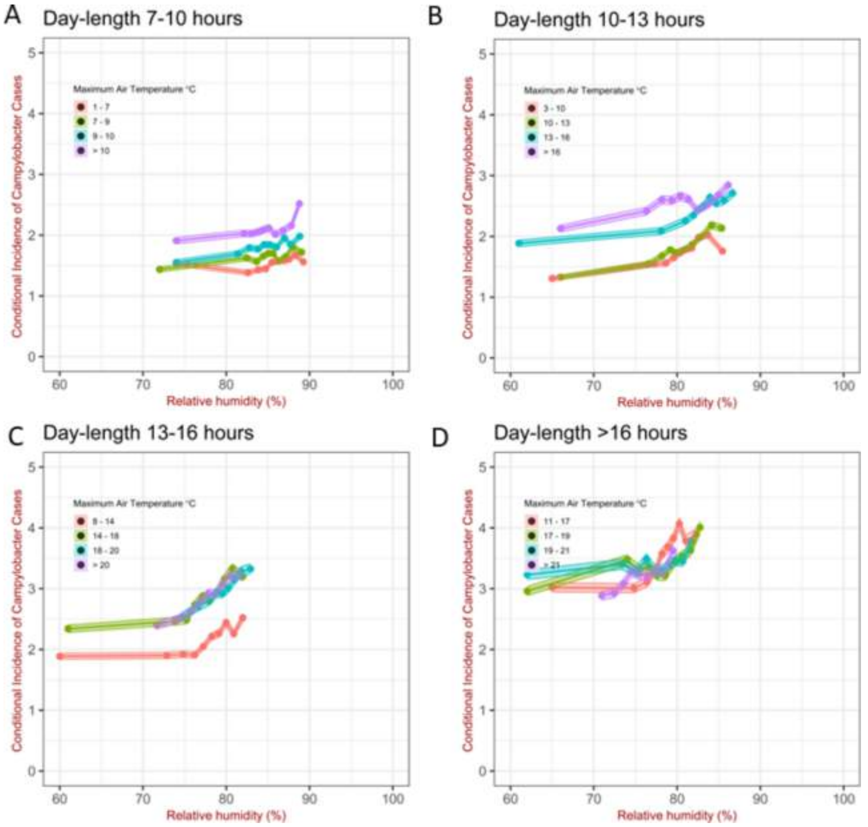

Fig K. As in figure 3, but it emphasizes the role of relative humidity by representing it on the $x - axis$.

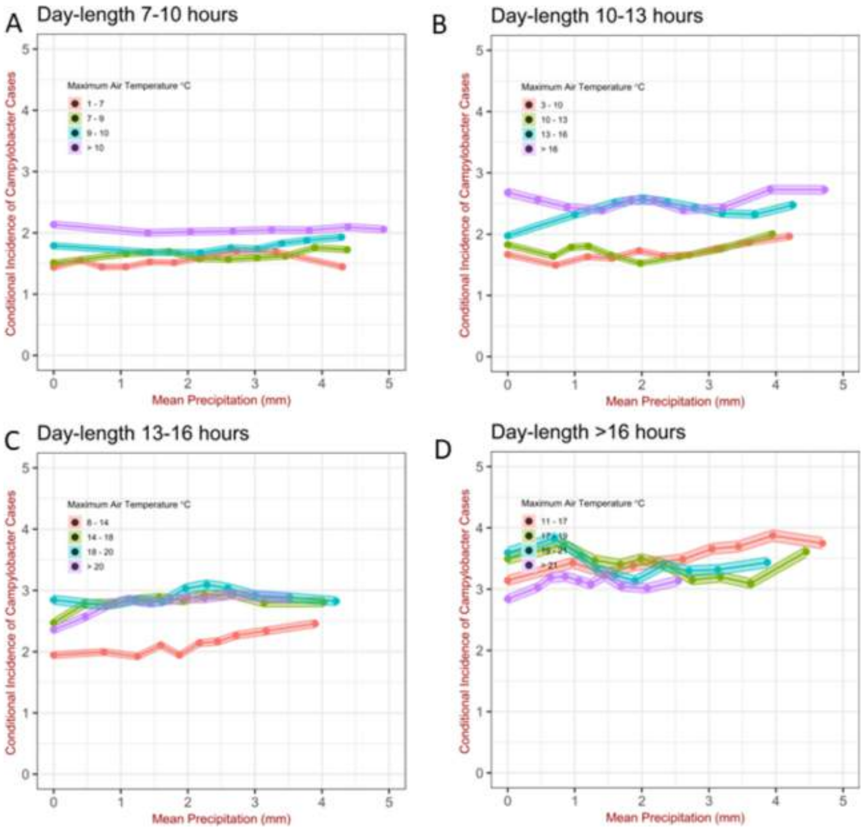

Fig L. As in figure 3 and K , but it emphasizes the role of rainfall by representing it on the $x - axis$.

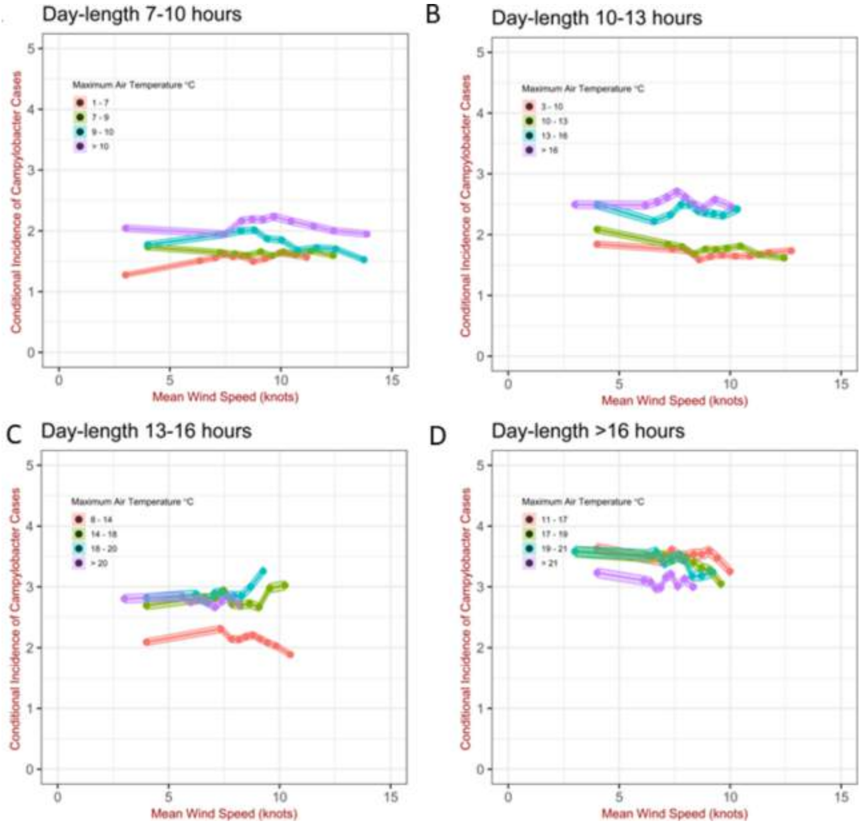

Fig M. As in figure 3 and K , but it emphasizes the role of mean wind speed by representing it on the $x - axis$.

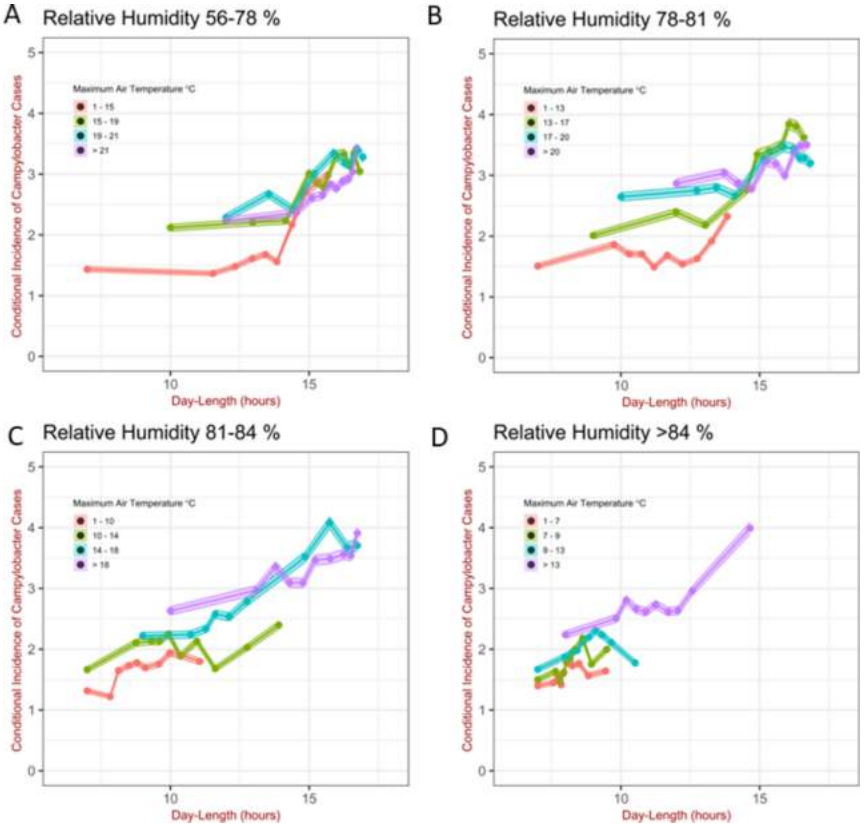

Fig N. As in figure 3 and K , but it emphasizes the role of mean day-length by representing it on the $x - axis$.

G Patterns in conditional incidence according to different weather variables (four weather factors simultaneously).

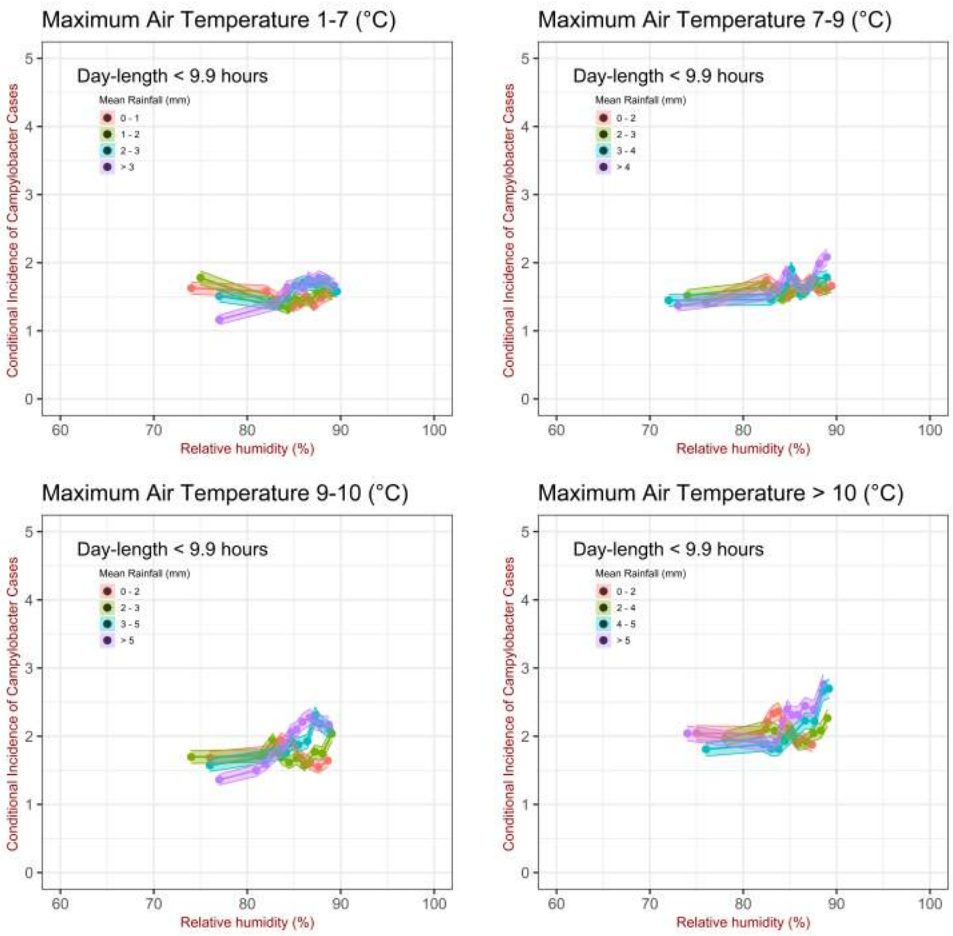

Fig O. Campylobacteriosis cases per $1,000,000$ per day conditioned to relative humidity stratified by rainfall, maximum air temperature and day-length; day-length <9.9 hours.

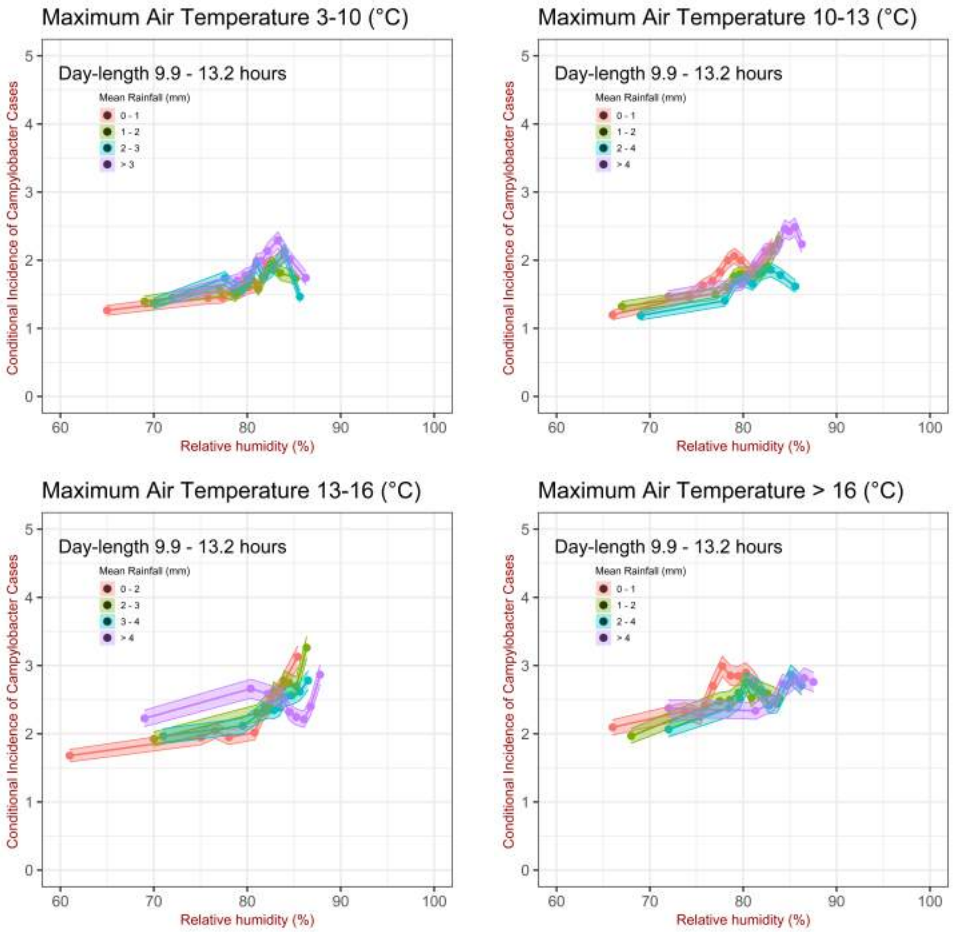

Fig O. Campylobacteriosis cases per $1,000,000$ per day conditioned to relative humidity stratified by rainfall, maximum air temperature and day-length; day-length > 9.9 and < 13.2 hours.

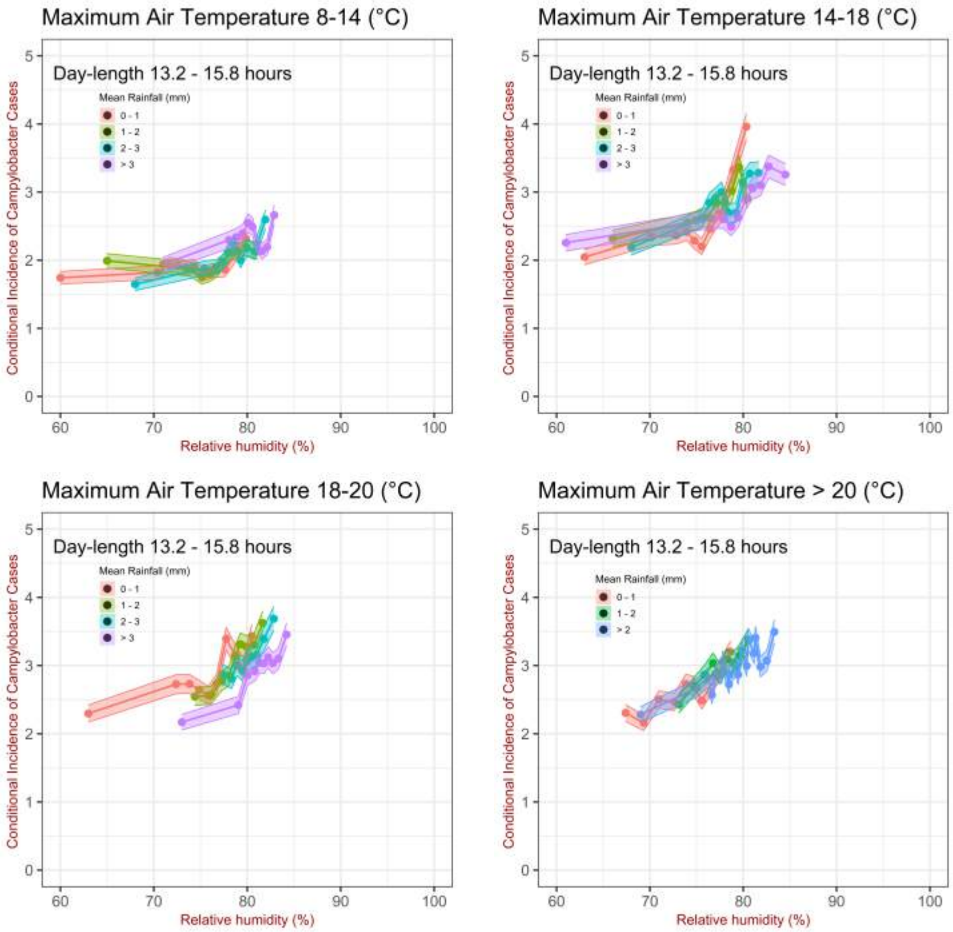

Fig O. Campylobacteriosis cases per $1,000,000$ per day conditioned to relative humidity stratified by rainfall, maximum air temperature and day-length; day-length > 13.2 and < 15.8 hours.

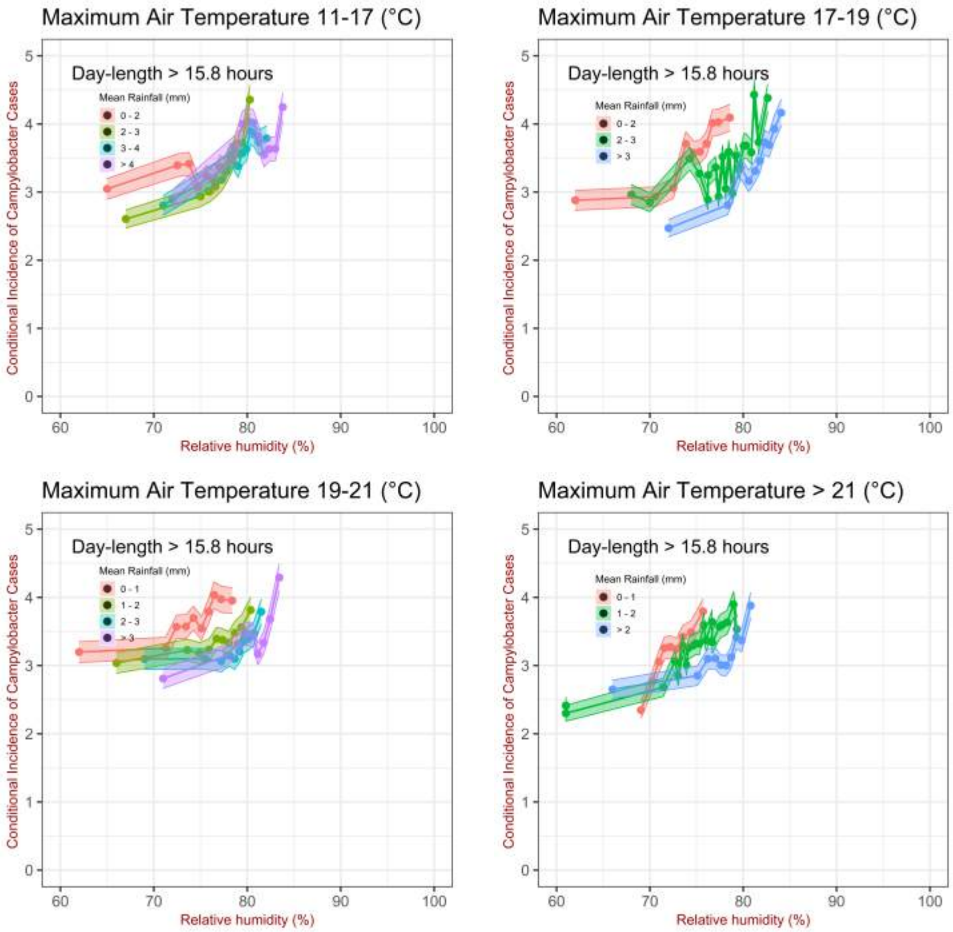

Fig O. Campylobacteriosis cases per 1, 000, 000 per day conditioned to relative humidity stratified by rainfall, maximum air temperature and day-length; day-length > 15.8 hours.

H Patterns in conditional incidence according to maximum air temperature and relative humidity for different periods of the year.

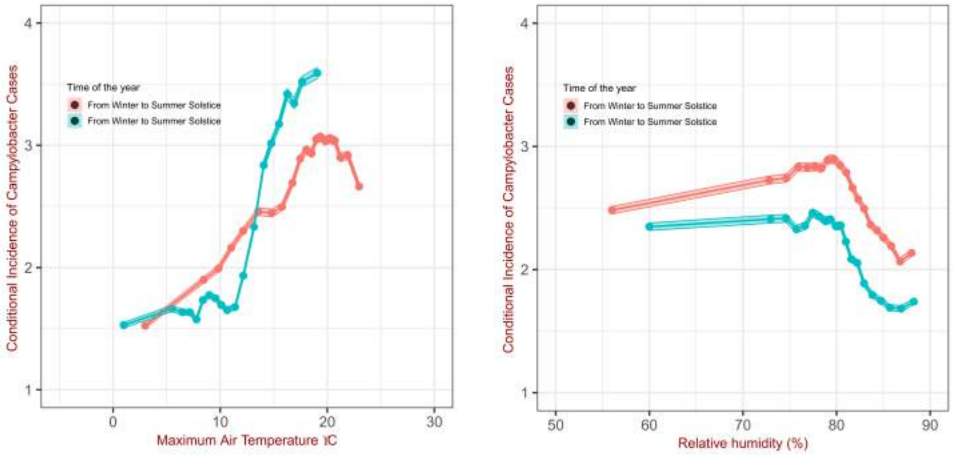

Fig P. Campylobacteriosis cases per $1,000,000$ per day conditioned to maximum air temperature and relative humidity for the time of the year between i) the shortest (winter solstice) and the longest (summer solstice) duration of day-length and between ii) the longest and the shortest duration of day-length. The choice allows comparisons at different months but ensures that the two parts of the year have the same distribution of day-length.

I Incidence of campylobacteriosis cases when the weather variables are averaged over different time-lags (three weather factors simultaneously).

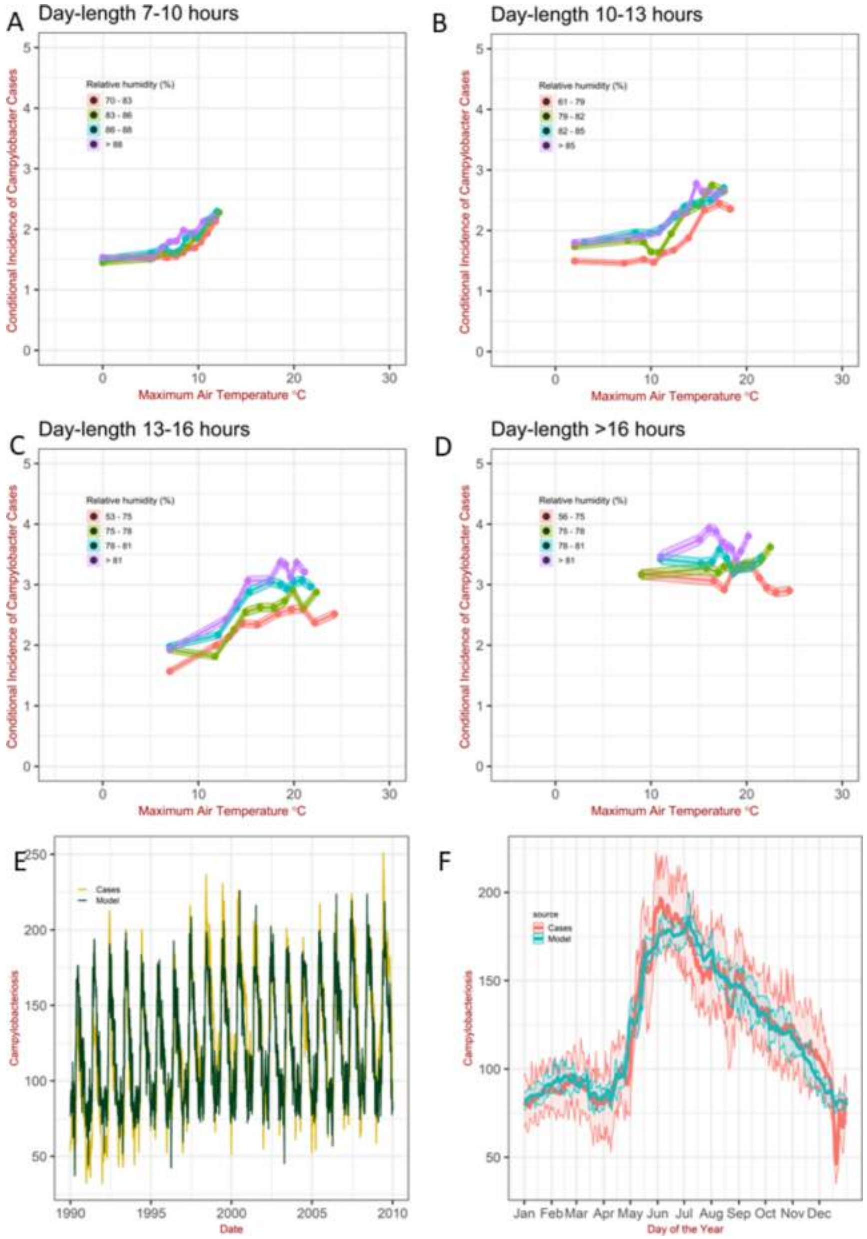

Fig Q. As in figure 3 and 4, but the weather variables were averaged over the past 7 days rather than 14 days.

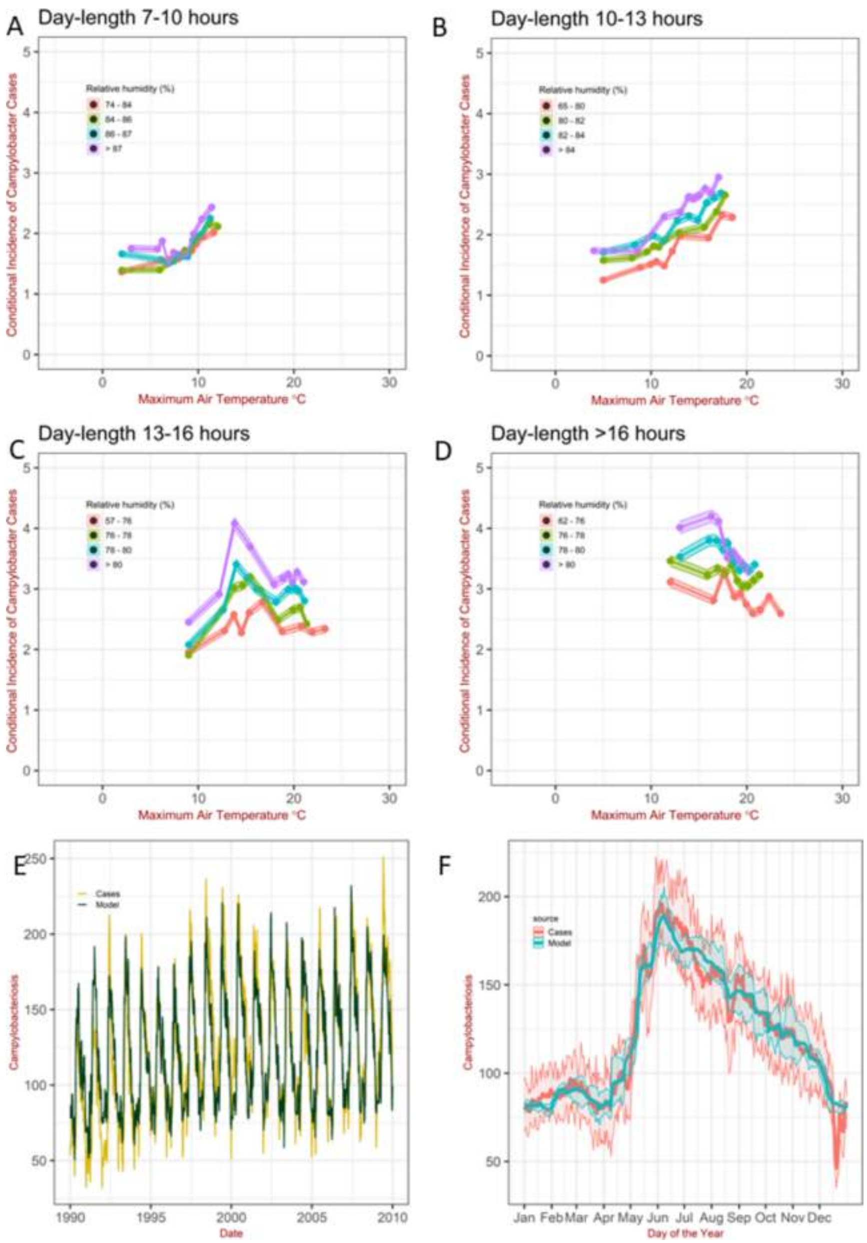

Fig R. As in figure 3 and 4, but the weather variables were averaged over the past 30 days rather than 14 days.

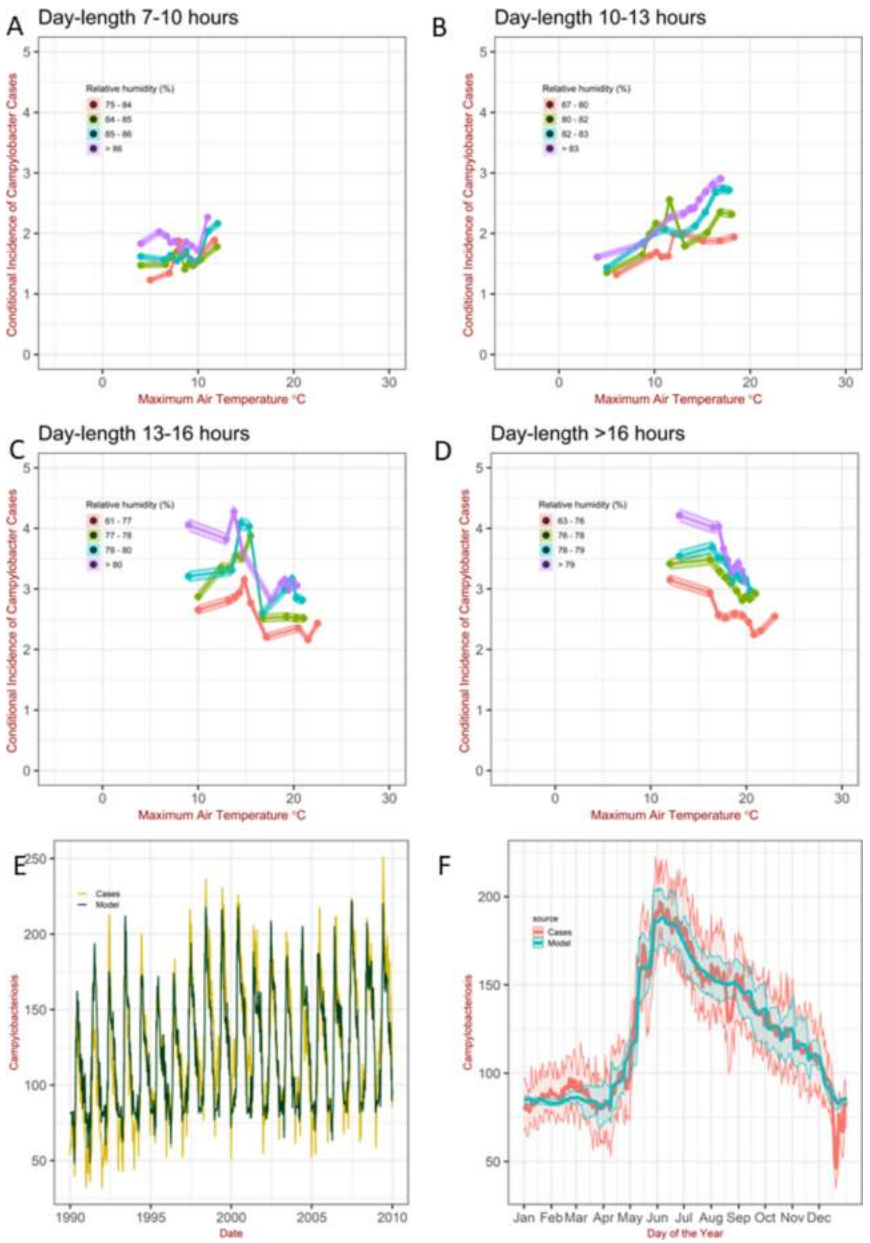

Fig S. As in figure 3 and 4, but the weather variables were averaged over the past 60 days rather than 14 days.

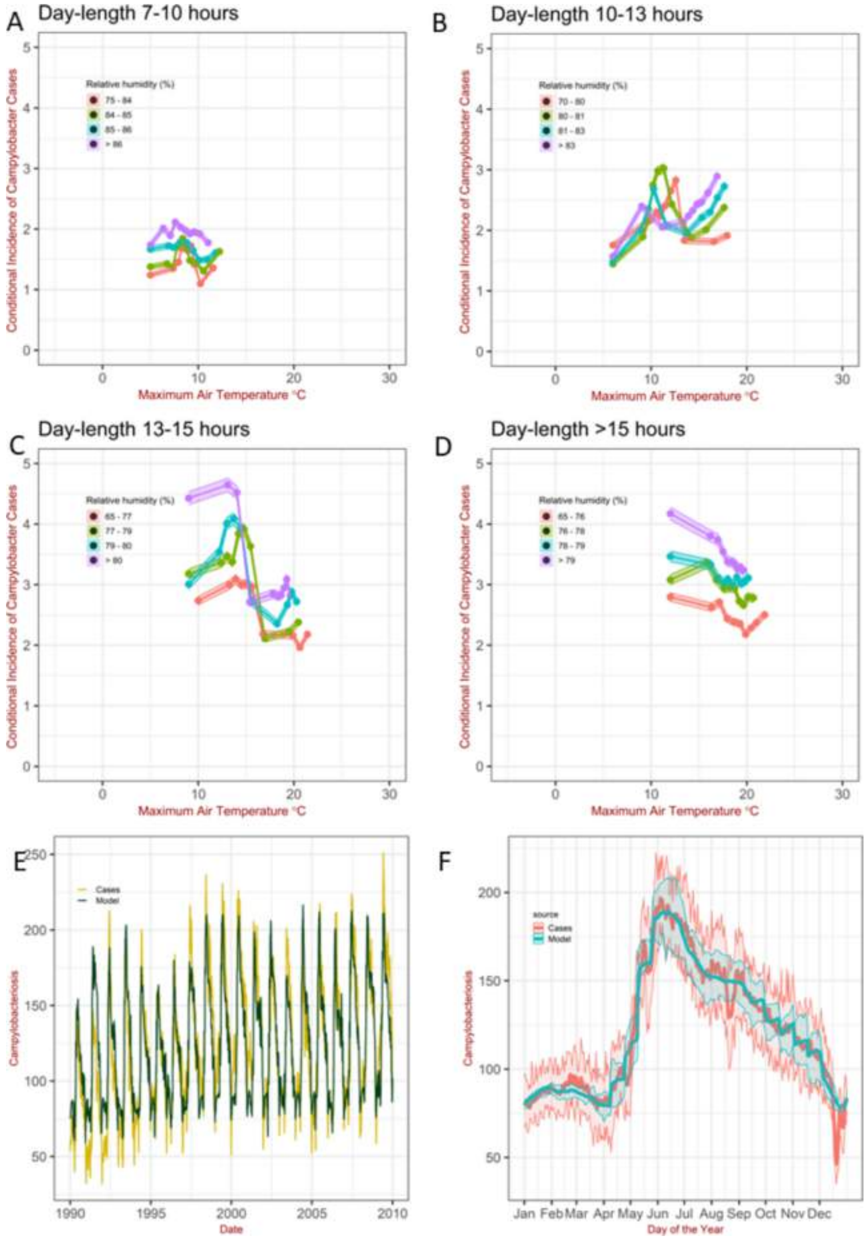

Fig T. As in figure 3 and 4, but the weather variables were averaged over the past 90 days rather than 14 days.

J Seasonal patterns for daily *Campylobacter* cases using only one predictor.

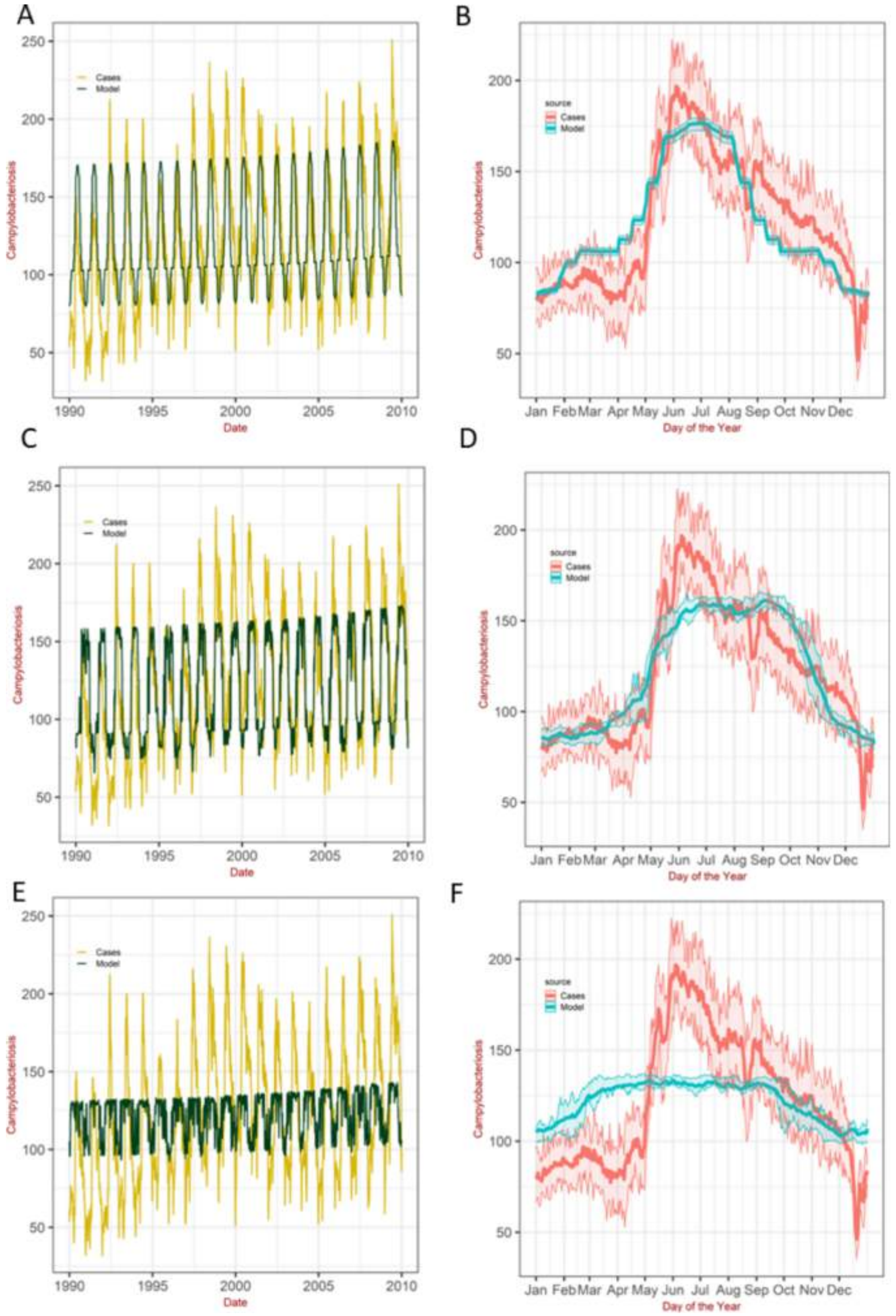

Fig U. Continued on next page

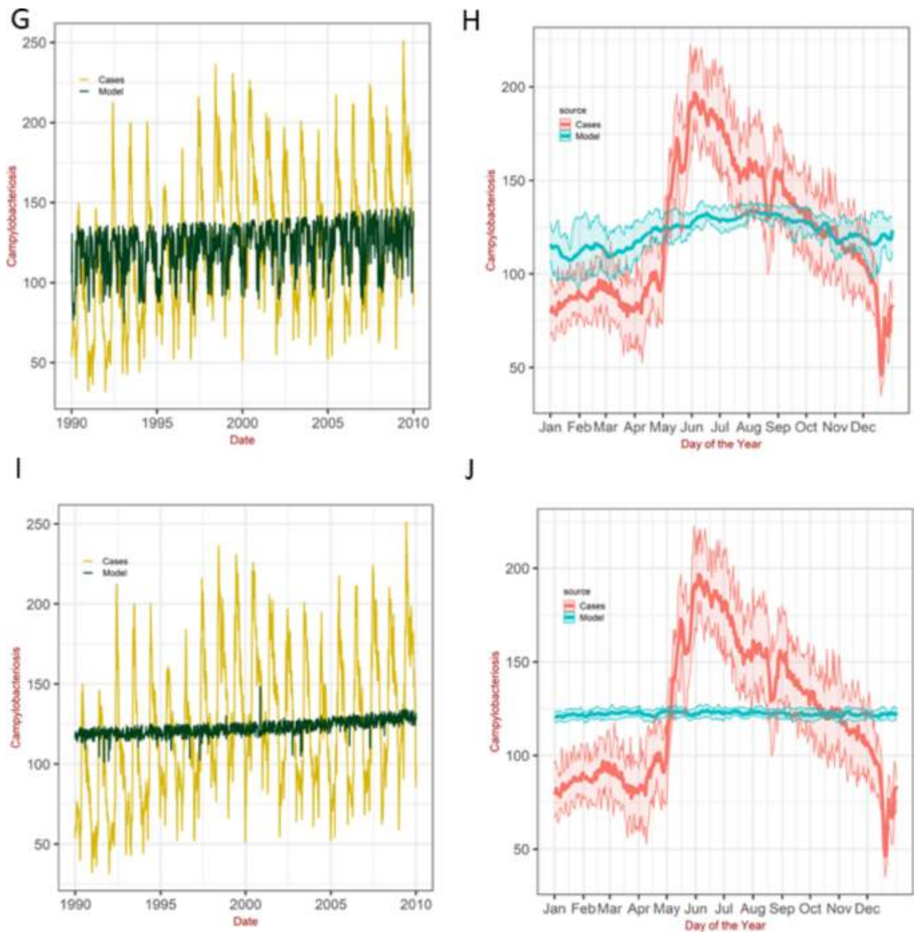

Fig U. As in Fig. 4.A and Fig. 4.B but using only one weather variable (averaged over the past 14 days) Panel A-B) day-length; Panel C-D) maximum air temperature; Panel E-F) relative humidity; Panel G-H) wind speed; Panel I-J) rainfall.

K Seasonal patterns for daily *Campylobacter* cases using only two predictors.

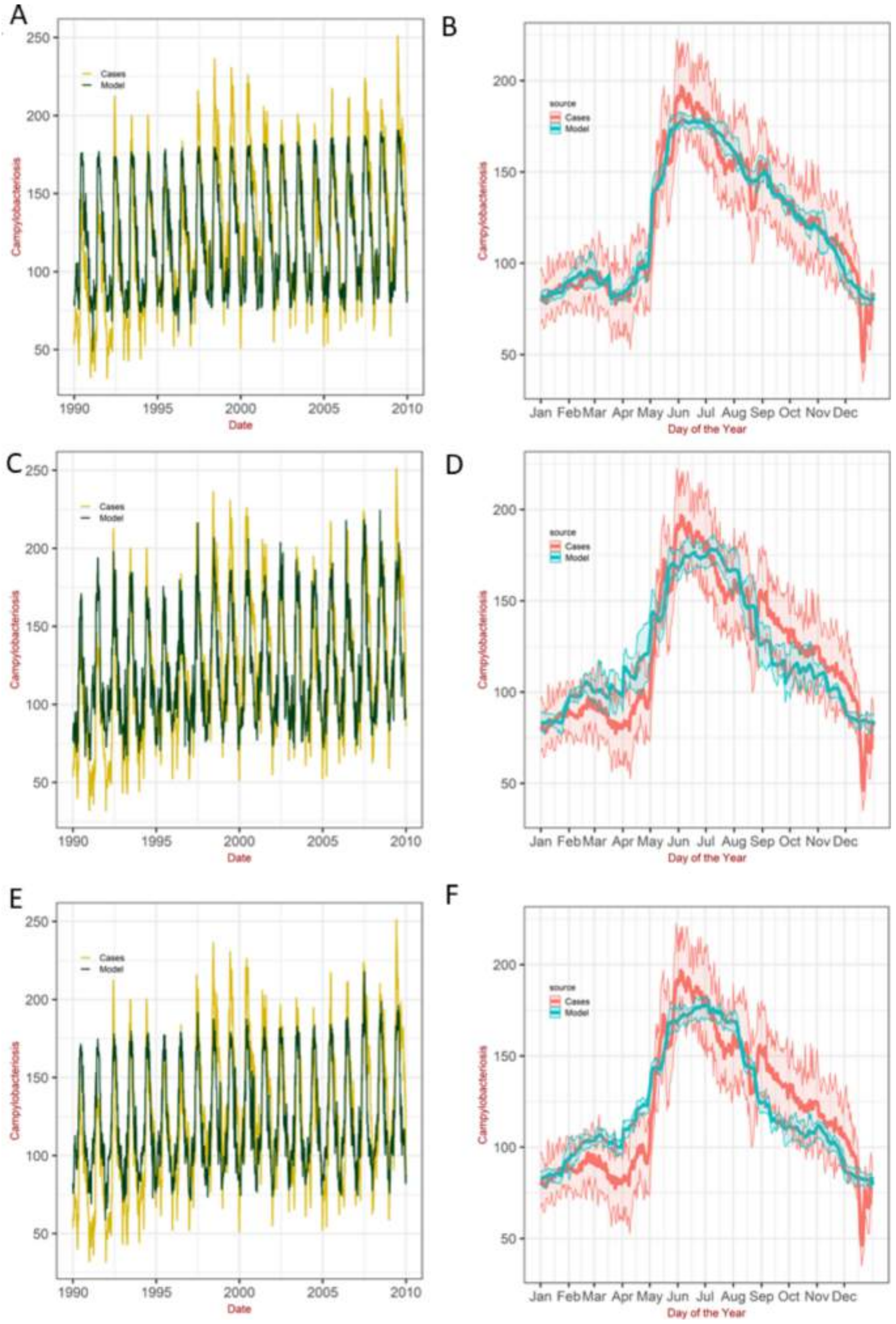

Fig V. Continued on next page

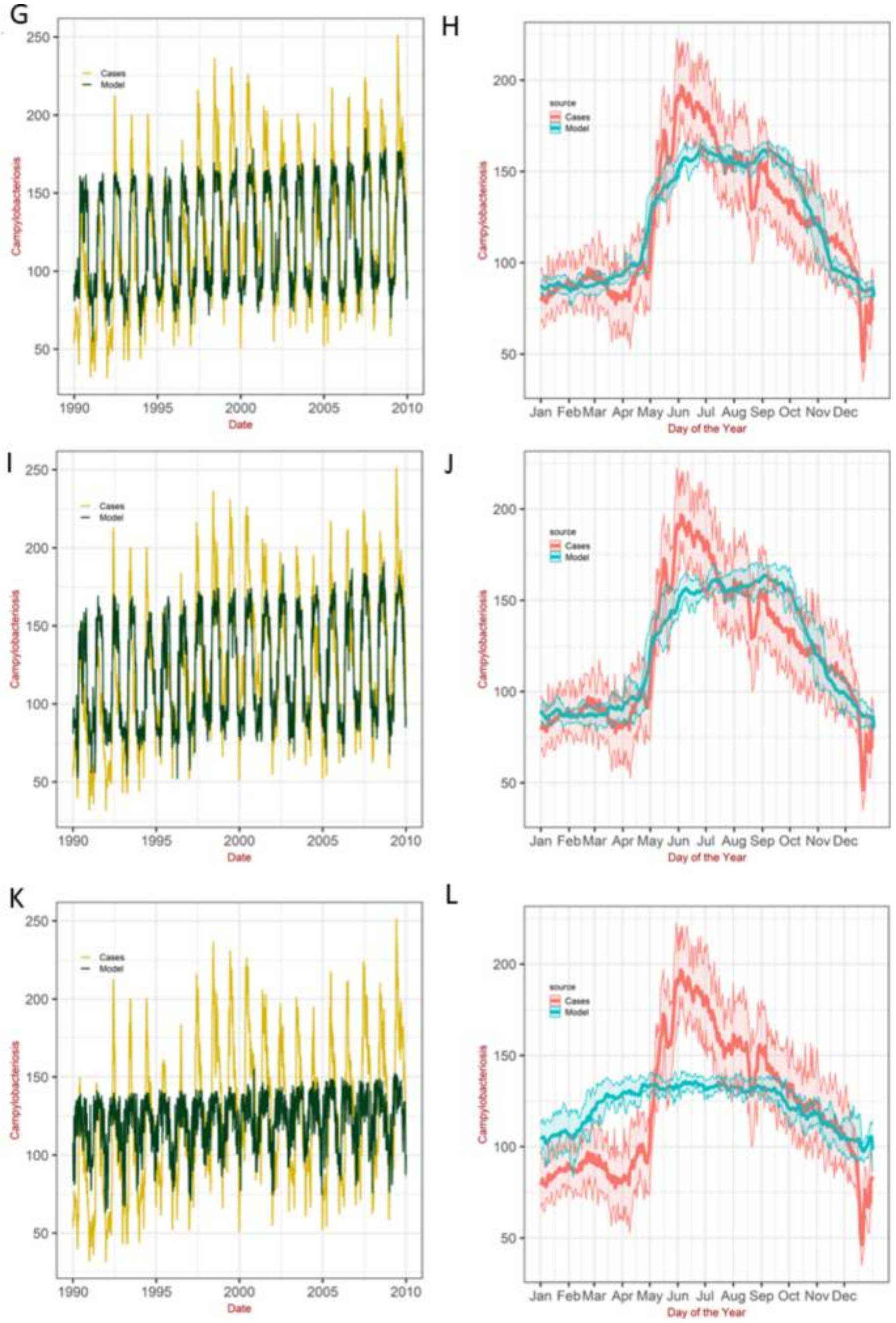

Fig V. As Fig. 4.A and Fig. 4.B but using only two weather variables (averaged over the past 14 days) Panel A-B) maximum air temperature and day-length; Panel C-D) relative humidity and day-length; Panel E-F) rainfall and day-length; Panel G-H) maximum air temperature and rainfall; Panel I-J) maximum air temperature and relative humidity; Panel K-L) relative humidity and rainfall.

L Predictions using rainfall, instead of relative humidity, as predictor.

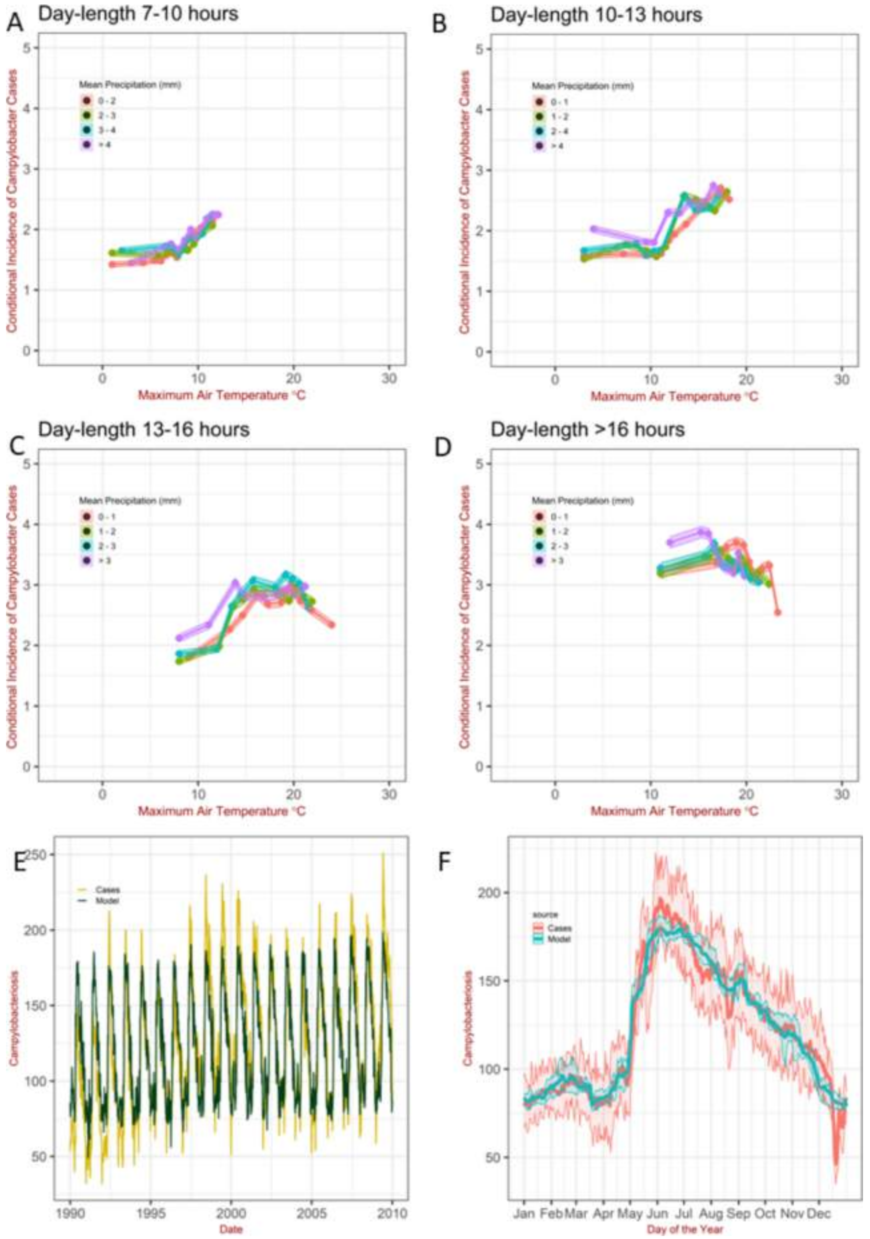

Fig W. A-D) As in figure 3. E-F) As in figure 4.A and 4.B but the weather variables are maximum air temperature, rainfall and day-length.

References

1. Public Health England. PHE centres: local authority lookup; 2014. Available from: `https://www.gov.uk/government/publications/phe-centres-local-authority-lookup`.

2. Djennad A, Lo Iacono G, Sarran C, Fleming LE, Kessel A, Haines A, et al. A comparison of weather variables linked to infectious disease patterns using laboratory addresses and patient residence addresses. BMC Infectious Diseases. 2018;18(1):198. doi:10.1186/s12879-018-3106-9.

3. Lo Iacono G, Armstrong B, Fleming LE, Elson R, Kovats S, Vardoulakis S, et al. Challenges in developing methods for quantifying the effects of weather and climate on water-associated diseases: A systematic review. PLOS Neglected Tropical Diseases. 2017;11(6):e0005659. doi:10.1371/journal.pntd.0005659.

4. Sartwell PE. The distribution of incubation periods of infectious disease. 1949. American journal of epidemiology. 1995;141(5):386–394; discussion 385.

5. Marinović AB, Swaan C, van Steenbergen J, Kretzschmar M. Quantifying reporting timeliness to improve outbreak control. Emerging infectious diseases. 2015;21(2):209–16. doi:10.3201/eid2102.130504.

6. Horn BJ, Lake RJ. Incubation period for campylobacteriosis and its importance in the estimation of incidence related to travel. Eurosurveillance. 2013;18(40):1–6.
