# Supplementary material for: A mathematical, classical stratification modeling approach to disentangling the impact of weather on infectious diseases: A case study using spatio-temporally disaggregated Campylobacter surveillance data for England and Wales
Source: PLoS Comput Biol. 2024 Jan 18;20(1):e1011714. doi: 10.1371/journal.pcbi.1011714 (PMC10796013; doi:10.1371/journal.pcbi.1011714)
Supplement: Attachment — Submitted filename: Response to Reviews report .pdf [file pcbi.1011714.s003.pdf]

Detailed responses to the points raised by the Reviewers.

We thank the Reviewers for the insightful and helpful comments. We have addressed specific comments below as well as made changes to the Revised Manuscript.

Please note,

i) some of the plots (predictions) have been updated to take into account that the reported temporal data were adjusted from day of year using a 7-day rolling mean to mitigate the under-reporting over bank holidays. The differences are negligible and we now clarify this in the text;

ii) the PLoS technical team identified materials that could be copyrighted. We have now uploaded the Publication Licence from Biorender (Figure 1) and indicated the source of the shapefiles (Fig 4.D). Figures S1 and S2 in the Supporting information were replaced with references to a previous publication (Djennad A, et al. 2018; doi:10.1186/s12879-018-3106-9) and to a webpage from Public Health England.

Reviewer #1: *The study aims to establish associations between epidemiological and weather data using the proposed metric, conditional incidence, which is thoroughly explained by the authors. This research provides valuable scientific evidence for understanding the mechanism of Campylobacter infections.*

We thank the reviewer for their supportive comments.

Reviewer #1: *One important consideration is whether the uncertainty associated with the conditional incidence can be quantified, similar to commonly used measures of association such as Pearson's correlation coefficient, odds ratio, or risk ratio. By estimating the uncertainty linked to the conditional incidence, it would greatly benefit future computational health research studies in assessing the impact of various environmental factors on disease cases.*

The uncertainty associated with the conditional incidence has been quantified by estimating confidence intervals. However, other measures can be considered. For example, as Colston, et al. (*GeoHealth*, *6*(1); 1–20, we can envisage a calculation of the relative risk conditioned to specific weather factors by dichotomising the continuous weather factors at adequate threshold (e.g., 8 degree for maximum air temperature) and calculating the relative risk; this would be the ratio of the (conditional) probability of campylobacteriosis for when the weather factor is above the chosen threshold (i.e., the "exposed group") to the probability of campylobacteriosis for when the weather factor is below the threshold (the "unexposed group"). In response to Reviewer comments, we now discuss this in section "Limitations and Future Work" in the revised manuscript.

Reviewer #1: *A minor correction is required in line number* ***196,*** *where "reconstructed the time-series" should be corrected to "reconstructed time series."*

In response to Reviewer comments, other minor corrections were addressed accordingly in the revised manuscript.

Reviewer #2: *This paper describes an innovative non-regression-based method to estimate the association between weather and risk of Campylobacter infection. The authors first investigated the most influential factors on the disease risk then performed a prediction analysis to compare with the observed incidences and seasonality. I enjoyed reading the paper. However, significant revisions are also needed before publication.*

We thank the Reviewer for the supportive comments.

Reviewer #2: *Line 66. How did the authors define and exclude travel-related cases?*

There is poor surveillance information available on travel related *Campylobacter* cases, but any individuals with recent travel reported on the request form were excluded from the analysis. In response to Reviewer comments, this is now clarified in the text.

Reviewer #2: *Line 67. The use of laboratory address was still a limitation of this study.*

The linkage was conducted on laboratory address rather than patient residential address on the basis that the discrepancies between some weather variables (temperature and rainfall) between the laboratory and the corresponding patient location are known to be small. We agree that this is a limitation in the study, however the choice presents some advantages: the laboratory address database was more complete than the patient residential address since the surveillance system had limited patient postcode records before 2008; the computational resources needed for data linkage at the individual patient postcode level can be significant large; and the use of laboratory address prevents challenges to comply with ethical and legal requirements of confidentiality. Furthermore, using patient residential address could introduce further bias as it is estimated that people spend up to 40% of time at locations other than their residence address (schools, workplace, transport).

In response to Reviewer comments, we now discuss this in section "Limitations and Future Work" in the revised manuscript.

Reviewer #2: *Now that the incubation period is 2-5 days and there some 1-4 days for reporting delay, why didn't the author use 7 days as the primary lag time for analysis?*

The reason is that the weather is not expected to have any influence after the pathogen has infected the host. Please note that the time-lag here was defined as "*the elapsed time during which the specific weather variables contribute to the occurrence of an infection.*" This is not a temporal gap during which the weather has no-effects. In response to Reviewer comments, we have clarified this in the revised manuscript.

Reviewer #2: *Lines 117-119. There indeed are other parameters (rainfall & wind speed) so they need to be stated in Methods.*

In response to Reviewer comments, we now state that the formalism can be readily adapted to rainfall and wind speed too in the revised manuscript.

Reviewer #2: *Last paragraph on page 4/29. D(j,x) should be D(t,x)?*

Thanks for spotting the typo; in response to Reviewer comments, this is corrected now.

Reviewer #2: *Page 5/29, all P(p,Campyl) should be Pr(p,Campyl) to be consistent.*

In response to Reviewer comments, this is corrected now. We changed all Pr(p,Campyl) to P(p,Campyl) instead to render the notation and the text less busy.

Reviewer #2: *Please explain OC in equation (1).*

OC was formally defined in the paragraph above, but in response to Reviewer comments, we now clarify that OC is the number of campylobacteriosis cases given the weather condition.

Reviewer #2: *Lines 140-143. I'm not following the rationale behind. As far as I can understand, if RH is an effect modifier, the plots of Pr(p,Campyl) should be a series of independent curves.*

We indeed agree with the Reviewer. However, perhaps the Reviewer misread the word *confounder* as *effect modifier*? The text indicates that if relative humidity is a confounder (not effect modifier) the plots are expected to collapse to the same curve, otherwise (i.e. effect modifier) the plots are expected to result in a series of independent curves. In response to Reviewer comments, we now state this.

Reviewer #2: *Lines 144-147, I'm also confused by the description. Can the authors give a very brief example?*

In response to Reviewer comments, we now explicitly show the quantiles in Figure S-6 B with explanation in the captions.

Reviewer #2: *"The expected number of daily infections is given by...see Fig S-3": I don't think this is relevant to the study since the authors didn't use R(inf) at all. Just explain the N(year,x) in the following equation.*

In response to Reviewer comments, the text has been modified as suggested.

Reviewer #2: *Line 175. I don't any specification on time lags in S1.*

In response to Reviewer comments, the text has been corrected.

Reviewer #2: *Line 178. Any reference for the equations? Why 15, 41, and 13 degrees? More importantly, results from ABM are synthetic disease data, as the authors have implied. Then how*

can this be used for validation of the methods the authors proposed? This directly lead to the question of validity of this approach. The authors should carefully justify it.

In response to Reviewer comments, this is now clarified in the text.

Reviewer #2: *Lines 187-188. Both figures in Fig 2 are ABM results, not from conditional incidence method.*

Following suggestion from Reviewer #3, this part is now in the Supplementary Material. This should remove any ambiguity in the Results/Methods section of the revised manuscript.

Reviewer #2: *Lines 195-198. As mentioned earlier, estimates from ABM are not real data either.*

What we mean here is that the method can identify the correct time-lag for real cases: what is needed is to try various time-lags and the one that fit the data better is likely the true time-lag.

Reviewer #2: *Line 203. There is no assessment in terms of rainfall and wind speed in Methods.*

In response to Reviewer comments, rainfall and wind speed are now mentioned in the Methods section of the revised manuscript.

Reviewer #2: *Lines 213-214. I don't think the association is apparent for the mainly focused time lag (14 days) of the entire manuscript . It only becomes obvious when the lag is over 30 days. Also, the authors should also note the negative association between 80-90% which should be discussed in the text. This is much more consistent and apparent than the positive association the authors mentioned for 75-80%.*

In response to Reviewer comments, we have rewritten this part of the revised manuscript. We have made it clearer that the analysis shows an association of the conditional incidence with relative humidity for day-length longer than 10 hours. We now clearly state that the peak around 75-80% becomes less evident when we increase the number of factors used in the stratification. We also state that depending on maximum air temperature, day-length and rainfall, the conditional incidence tends to increase with relative humidity either monotonically or reaching a peak in the region of high relative humidity followed by a decrease; we believe, this is what the Reviewer refers to when they mention a negative association between 80-90%.

Reviewer #2: *Lines 225-226. I think the authors should explore more on this negative association which seems consistent across time lags, rather than speculating it as a spurious relationship.*

The Reviewer should refer to the decrease of the conditional incidence with wind speed. When we observed the decrease, we thought that this was a strong indication of the fly hypothesis (i.e., that campylobacteriosis is mechanically transmitted by flies (*musca domestica)*). This because flies' activity is reduced at high wind speed and therefore decrease incidence. However, after stratifying the incidence with higher number of weather factors the negative association is significantly

reduced (Figure S.13), this is the key point of stratifying with higher number of factors. Also, wind speed tends to be lower for longer day-length and for high maximum temperature (Fig. S.7), which are the conditions when the incidence tend to be high. Therefore, we think that the negative association is due to correlation of wind speed with other factors like day-length and maximum temperature. Still, the fly hypothesis cannot be dismissed as in houseflies ambient temperature has a direct influence on the physical activity and the life span and flight activity increases with temperature. In response to Reviewer comments, we briefly discuss this in the revised manuscript.

Reviewer #2: *Line 226. Should also mention Fig 4F.*

In response to Reviewer comments, corrected.

Reviewer #2: *Fig S15 is missing in the result text. The author should re-organize the text to make sure all figures appear in order in the text.*

In response to Reviewer comments, corrected.

Reviewer #2: *Lines 238-239. An analysis similar to what? Need to re-organize the result.*

In response to Reviewer comments, corrected.

Reviewer #2: *Lines 247-248. Why not? The authors have already found significant variations across time , not like rainfall and wind speed.*

We agree with the Reviewer and acknowledge this in the revised manuscript. In response to Reviewer comments, we have now clarified that 3 factors are sufficient to make accurate predictions; and therefore time of the year was not included on parsimony ground.

Reviewer #2: *Lines 263-264. The authors should discuss why in conditional incidence approach rainfall seems not to be associated with the cases but in prediction it serves as a necessary component? Or is it because that actually only day-length (with or without maximum temperature) is the most essential element predicting the incidence? This can be easily tested.*

In response to Reviewer comments, we have extensively tested and different combinations of weather factors separately. See Figure S23, S24 and related discussion.

Reviewer #2 *Lines 265-279. I'm not following. I understand that if only one variable was used with the other two constant (Fig 7A, B, and C), they can not capture the variation of the seasonality. But how can these three individual analysis elucidate that the estimates from Fig 6B are the results originated from the combination of all three variables?*

The only non-constant variable is still seasonal; and therefore we can look at individual effect for the fixed level of the other two factors. In response to Reviewer comments, we have slightly changed the text to clarify this.

Reviewer #2: Lines 288-296. This should be explicitly somewhere in Methods and Results for the readers to better understand the results and figures (and the motivation behind them).

In response to Reviewer comments, we now explain in Methods and Results in the revised manuscript that we consider situations when we used two or one explanatory variables.

Reviewer #2: *Lines 336-338. Can the authors elaborate more on the comparison between this approach and case crossover? Since it's not a regression-based approach, how are other baseline characteristics be controlled in the analysis?*

In response to Reviewer comments, we decided to remove this part.

Reviewer #2: Limitations should be discussed. And there should be conclusions.

In response to Reviewer comments, we have added a Limitation subsection and a Conclusion section in the revised manuscript.

Reviewer #3: *Congratulations on an excellent analysis that [..] PLOS Computational Biology.*

We thank the Reviewer for the supportive comment.

Reviewer #3: *Title. I suggest rewording the title to better reflect the aim and design of the study and the data sources used. For example: "A mathematical, classical stratification modeling approach to disentangling the impact of weather on infectious diseases: a case study using spatiotemporally disaggregated Campylobacter surveillance data for England and Wales."*

We agree with the Reviewer; and we changed the title in the revised manuscript as suggested.

Reviewer #3: *Abstract. I suggest simplifying the take-away statement to "The predicted incidence of campylobacteriosis increased by 1 case per million for every 5 degree increase in temperature within the range of 8 – 15 degrees. No association was observed outside that range" and making a similar statement for humidity which quantifies the effect size found by the model.*

In response to Reviewer comments, we have modified the abstract as suggested.

Reviewer #3: *Background. I'm not sure that the authors are always citing the most relevant literature. For the statement about the global burden of diarrheal disease (#2) consider citing https://pubmed.ncbi.nlm.nih.gov/34416195/ and/or https://pubmed.ncbi.nlm.nih.gov/27839855/. For the statement about environmental impact on pathogens (#6) consider citing https://pubmed.ncbi.nlm.nih.gov/35968032/. In the second paragraph, consider citinghttps://pubmed.ncbi.nlm.nih.gov/35024531/, a study which tackles most of the fundamental issues that the authors highlight.*

We thank the reviewer for the suggestion. In response to Reviewer comments, we now cite the recommended literature, as well as other articles, in the background and throughout the revised manuscript.

Reviewer #3: *I am not familiar with the term "phenomenological" used in this context and I found it a little jarring, though this may be a technical use of the term with which I am not familiar.*

By phenomenological we simply meant a description of how the incidence "looks like" without discussing the causes. In response to Reviewer comments, we have removed the word for simplicity in the revised manuscript.

Reviewer #3: *Please state the aim of the paper more clearly. Is it to quantify the effects of weather on Campylobacter, or is it to showcase a methodology that can be adapted to other diseases and contexts? There should be a sentence that starts "The aim of this analysis is to…"*

In response to Reviewer comments, the text is corrected as suggested.

Reviewer #3: *Please state the units of weather variables. Were total daily rainfall volumes used or averages?*

In response to Reviewer comments, we have now included the units.

Reviewer #3: *I assume that campylobacteriosis was diagnosed in stool samples, but this is not stated anywhere. What diagnostic method was used (culture? PCR?), and was it standard across laboratories?*

In response to Reviewer comments, this has now been clarified in the text of the revised manuscript.

Reviewer #3: *The authors explain that cases were georeferenced to the laboratory locations, and not residences due to completeness considerations. However, they don't explain why they used the date that the samples arrived at the laboratory and not the date when they were collected from the patient. Was this information similarly incomplete? If sample collection date could be used, it would eliminate the need to incorporate reporting delays.*

Unfortunately, the date when they were collected from the patient was not available; thus we have to rely on the date that the samples arrived at the laboratory and therefore the reporting delays.

In response to Reviewer comments, this is now clarified in the text of the revised manuscript.

Reviewer #3: *I do not understand what is meant by "We restricted our analysis to cases when the infection likely occurred … between the 1 January 1990 and 31 December 2009 to ensure … that all postcodes were represented."*

We have cases up to April 2010 but this would bias the seasonal patterns. Also, we have cases before 1 January 1990 but for a limited number of postcodes. As we were not sure that all postcode started the surveillance scheme exactly at the same time; therefore to be cautious, we started the analysis at 1 January 1990.

Reviewer #3: *Instead of generating a random realization of the incubation period and reporting delay, consider aggregating variables over a lagged window of exposure (see https://pubmed.ncbi.nlm.nih.gov/36796984/).*

According to https://pubmed.ncbi.nlm.nih.gov/36796984/ and the original work they cite (Geohealth, 6 (2022), Article e2021GH000452), hydrometeorological exposures were aggregated over a 7-day moving time window from 3 to 9 days prior to the date of sample collection. This means that the incubation period and reporting delay are treated as a fixed 3 day time-lag. Although this would have been a simpler procedure, the random realization we use introduced a more realistic variability. In response to Reviewer comments, we now clarify this in the text. Furthermore, from preliminary tests we have observed that the findings are not sensitive to the particular stochastic realization; and therefore, we expect that aggregating variables over a lagged window would not sensibly change the findings.

Reviewer #3: *The justification for including day length as an explanatory variable is not clear to me. As is apparent in figure 7F, this variable has a very smooth, sinusoidal distribution. It strikes me that the effect of including it will therefore be similar to that of including a harmonic term/ Fourier function (https://pubmed.ncbi.nlm.nih.gov/10396550/), i.e., an adjustment for laboratory catchment area-specific seasonality patterns so that the effects of the other variables can be interpreted as short-term, interdiurnal associations (see https://pubmed.ncbi.nlm.nih.gov/31229000/). If this is the case, then this should be stated explicitly, along with a justification for only using just one term, when the seasonal pattern of Campylobacter incidence is irregular (fig S-5). If this is not the case, and the authors instead hypothesize some direct association between day length and the infection outcome (such as the cumulative effect of solar radiation either on the bacteria itself or on host immunity), then this too should be stated. Either way, it is unsurprising that this variable should show an association with disease incidence, since it is essentially a proxy for the seasonal cycle.*

Our initial goal was to ascertain if the observed seasonality in the incidence was entirely driven by the seasonality in the weather factors due to the response of the pathogen to some environmental stimuli. For instance, if the incidence is driven solely by survival of pathogen in the environment, the incidence is expected to depend on factors like temperature, relative humidity and ultraviolet radiation (UV). We tested the model with temperature and relative humidity and there are clearly discrepancies between predictions and empirical data (Fig. S24).

Ideally, we would include the effect of UV, but because the lack of such data, we used *daylength* as proxy. Daylength is described only by a trigonometric function depending only on time of the year and latitude ("A Model Comparison for Daylength as a function of latitude and day of the year", Ecological Modelling, volume 80 (1995)).

Thus, we agree with the Reviewer that the effect of including daylength might simply results in the inclusion of some harmonic terms. We performed further tests (also in response to Reviewer #2 comments), and the analysis clearly shows that daylength alone (either as proxy for the seasonal cycle or UV) is not sufficient to explain the empirical patters, as this would results in a symmetric distribution of the incidence in line with the symmetric distribution of daylength. Similar

discrepancies are observed if we combine daylength and relative humidity and daylength and rainfall (Fig S23 and S24).

We can also infer that time of the year, has an effect on the incidence, even if the daylength is the same, as shown in Fig. S18.

Taken together, the research shows that the seasonality of campylobacteriosis is not driven by cyclic nature of daylength alone. The current analysis, however, cannot distinguish how and if daylength is involved in the causal pathways; for instance, daylength could be a proxy of for the effect of radiation on the bacterium or representing periodic human behaviour driven by the seasonal cycle of daylength. To clearly address this point, we should stratify the data by daylength and a measure of radiation, e.g. UV, and ascertain if daylength or UV are actual predictors. This can be left for future research. In response to Reviewer comments, it is now stated in the model limitation in the revised manuscript.

Reviewer #3: *Please narrow the range of the axes in Figure 5 so that the lines occupy more of the plot space and the shape of the associations will be more discernable.*

In response to Reviewer comments, we have now reduced the range of the axes as suggested.

Reviewer #3: *Compare the results with Colston and colleagues (https://pubmed.ncbi.nlm.nih.gov/35024531/) who likewise found a strong, non-linear association of Campylobacter with temperature - though with an upper plateau at a much higher temperature – and negligible effects of wind speed and rainfall, but did not find an association with relative humidity in a model that adjusted for soil moisture.*

We thank the Reviewer for pointing to Colston *et al.* paper. In response to Reviewer comments, we now discuss the differences between the two studies in the revised manuscript. About the association with relative humidity, the model of Colston *et al.* comprised all exposures and covariate variables including soil moisture and relative humidity. In our work, the data on soil moistureis correlated with atmospheric humidity were not available; and therefore we could not assess if relative humidity is a true predictor or simply a proxy for soil moisture.

Reviewer #3: *I am confused by the comment that this analysis resembles a case-crossover study design.*

In response to Reviewer comments, we have removed this part.

Reviewer #3: *An additional limitation of this study is that the data comes from a high-income, temperature latitude and low Campylobacter-burden context and therefore, it may not be possible to extrapolate these findings to the low-resource, tropical settings where incidence of the disease and its sequelae are highest.*

In response to Reviewer comments, whave added this to the Discussion section of the revised manuscript.

Reviewer #3: *I do not fully understand the ABM part of the analysis. I think the justification for this needs to be made more explicit but, since it seems to be some sort of validation exercise, why are the results presented so early on (figs 2 and 3)? As a reader, I want to see the actual results of the model first before seeing proof of its validity, which is of secondary interest. Can Figures 2 and 3 not be relegated to the supplementary materials? There are already a lot of figures in the main article.*

In response to Reviewer comments, we have moved this part to Supplementary Materials section of the revised manuscript.

Reviewer #3: *I suggest keeping the background, methods, results, and discussion more distinct throughout. For example, the first few sentences of the "analysis methods" section reads like background and cites background literature.*

In response to Reviewer comments, we have rearranged part of the text.
