# Supplementary material for: A mathematical, classical stratification modeling approach to disentangling the impact of weather on infectious diseases: A case study using spatio-temporally disaggregated Campylobacter surveillance data for England and Wales
Source: PLoS Comput Biol. 2024 Jan 18;20(1):e1011714. doi: 10.1371/journal.pcbi.1011714 (PMC10796013; doi:10.1371/journal.pcbi.1011714)
Supplement: Attachment — Submitted filename: review report.docx [file pcbi.1011714.s002.docx]

The study aims to establish associations between epidemiological and weather data using the proposed metric, conditional incidence, which is thoroughly explained by the authors. This research provides valuable scientific evidence for understanding the mechanism of Campylobacter infections.

One important consideration is whether the uncertainty associated with the conditional incidence can be quantified, similar to commonly used measures of association such as Pearson's correlation coefficient, odds ratio, or risk ratio. By estimating the uncertainty linked to the conditional incidence, it would greatly benefit future computational health research studies in assessing the impact of various environmental factors on disease cases.

A minor correction is required in line number **196,** where "reconstructed the time-series" should be corrected to "reconstructed time series."
